# Approaching Inflammation Paradoxes—Proinflammatory Cytokine Blockages Induce Inflammatory Regulators

**DOI:** 10.3389/fimmu.2020.554301

**Published:** 2020-10-19

**Authors:** Ming Liu, Jason Saredy, Ruijing Zhang, Ying Shao, Yu Sun, William Y. Yang, Jirong Wang, Lu Liu, Charles Drummer, Candice Johnson, Fatma Saaoud, Yifan Lu, Keman Xu, Li Li, Xin Wang, Xiaohua Jiang, Hong Wang, Xiaofeng Yang

**Affiliations:** ^1^ Centers for Cardiovascular Research, Inflammation, Translational & Clinical Lung Research, Lewis Katz School of Medicine at Temple University, Philadelphia, PA, United States; ^2^ Department of Cell Biology and Genetics, School of Basic Medical Science, Shanxi Medical University, Taiyuan, China; ^3^ Centers for Metabolic Disease Research, Cardiovascular Research, & Thrombosis Research, Lewis Katz School of Medicine at Temple University, Philadelphia, PA, United States; ^4^ Department of Nephrology, The Affiliated People’s Hospital of Shanxi Medical University, Taiyuan, China; ^5^ Rutgers University, New Brunswick, NJ, United States; ^6^ Department of Cardiology, The Affiliated People’s Hospital of Shanxi Medical University, Taiyuan, China; ^7^ Department of Rheumatology, The Second Hospital of Shanxi Medical University, Taiyuan, China; ^8^ Departments of Pharmacology, Microbiology and Immunology, Lewis Katz School of Medicine at Temple University, Philadelphia, PA, United States

**Keywords:** proinflammatory cytokine blockage, proinflammatory cytokines, inflammation, innate immune regulators, reactive oxygen species

## Abstract

The mechanisms that underlie various inflammation paradoxes, metabolically healthy obesity, and increased inflammations after inflammatory cytokine blockades and deficiencies remain poorly determined. We performed an extensive –omics database mining, determined the expressions of 1367 innate immune regulators in 18 microarrays after deficiencies of 15 proinflammatory cytokines/regulators and eight microarray datasets of patients receiving Mab therapies, and made a set of significant findings: 1) proinflammatory cytokines/regulators suppress the expressions of innate immune regulators; 2) upregulations of innate immune regulators in the deficiencies of IFNγ/IFNγR1, IL-17A, STAT3 and miR155 are more than that after deficiencies of TNFα, IL-1β, IL-6, IL-18, STAT1, NF-kB, and miR221; 3) IFNγ, IFNγR and IL-17RA inhibit 10, 59 and 39 proinflammatory cytokine/regulator pathways, respectively; in contrast, TNFα, IL-6 and IL-18 each inhibits only four to five pathways; 4) The IFNγ-promoted and -suppressed innate immune regulators have four shared pathways; the IFNγR1-promoted and -suppressed innate immune regulators have 11 shared pathways; and the miR155-promoted and -suppressed innate immune regulators have 13 shared pathways, suggesting negative-feedback mechanisms in their conserved regulatory pathways for innate immune regulators; 5) Deficiencies of proinflammatory cytokine/regulator-suppressed, promoted programs share signaling pathways and increase the likelihood of developing 11 diseases including cardiovascular disease; 6) There are the shared innate immune regulators and pathways between deficiency of TNFα in mice and anti-TNF therapy in clinical patients; 7) Mechanistically, up-regulated reactive oxygen species regulators such as myeloperoxidase caused by suppression of proinflammatory cytokines/regulators can drive the upregulation of suppressed innate immune regulators. Our findings have provided novel insights on various inflammation paradoxes and proinflammatory cytokines regulation of innate immune regulators; and may re-shape new therapeutic strategies for cardiovascular disease and other inflammatory diseases.

## Introduction

Cardiovascular diseases (CVDs), which include coronary heart disease, hypertension, stroke, and peripheral artery disease, collectively comprise the number one cause of death globally (1, 2). Our and others’ recent reports showed that CVD stressors and risk factors such as hyperlipidemia (3, 4), hyperglycemia (5), hyperhomocysteinemia (6, 7), and chronic kidney disease (8–10), promote atherosclerosis and vascular inflammation *via* several mechanisms. These mechanisms include endothelial cell activation (3, 11–14) and injury (15); caspase-1/inflammasome activation (8, 10), mitochondrial reactive oxygen species (ROS) (4); differentiation of Ly6C^high^ mouse monocytes and CD40^+^ human monocytes (7, 16–18); decreased/transdifferentiated CD4^+^Foxp3^+^ regulatory T cells (Treg) (19–22); impaired vascular repairability of bone marrow-derived progenitor cells (23, 24); downregulated histone modification enzymes (25) and increased expressions of trained immunity pathway enzymes (26). These reports have clearly demonstrated that inflammation mechanisms play significant roles in the initiation and pathogenesis of vascular inflammation and atherosclerosis.

Proinflammatory cytokines (PCs) are key regulators of inflammation, participating in acute (27) and chronic inflammation *via* a complex and sometimes seemingly contradictory network of interactions (28). Numerous reports of gene deficiency within mouse models showed that while PCs promote vascular inflammation and atherosclerosis; deficiencies of these cytokine genes lead to decreased atherosclerosis. In contrast, several anti-inflammatory cytokines inhibit vascular inflammation and atherosclerosis, and the deficiencies of those anti-inflammatory cytokine genes result in increased inflammation and atherosclerosis (29). This recent progress led to the development of many cytokine blockage-based therapies for inflammatory diseases and CVDs. The CANTOS trial with the monoclonal antibody (Mab) Canakinumab to block proinflammatory cytokine interleukin-1β (IL-1β) was a recent success in treating coronary artery disease (30). However, recent reports from our and others’ teams suggest that inhibition of one or more proinflammatory regulators such as cytokines or microRNAs (miRs) can lead to new waves of inflammation. In our previous studies, deficiency of proinflammatory microRNA-155 (miR155) in atherogenic apolipoprotein E knock-out (ApoE KO, or ApoE^-/-^) mice results in the establishment of the first metabolically healthy obesity (MHO) mouse model with decreased aortic atherosclerosis, increased obesity, white adipose tissue hypertrophy and non-alcoholic fatty liver disease but without insulin resistance (31). In another report we showed that, analyzing 109 microRNAs (miRs) reported in four hyperlipidemia-related diseases (HRDs) such as atherosclerosis, non-alcoholic fatty liver disease (NAFLD), obesity, and type II diabetes (T2DM), we found that miR155 and miR221 are significantly modulated in all four HRDs. We hypothesized that miR155 is a proinflammatory, proatherogenic but obesity-suppressed master regulator. Deficiency of miR155 results in a “second wave of inflammation” in the high-fat feeding MHO model, which is our proposed new concept. Indeed, our results showed that high-fat feeding leads to a new “second wave of inflammation”, as we termed, in miR155^-/-^/ApoE^-/-^ MHO mice with increased plasma proinflammatory adipokines leptin and resistin in plasma and white adipose tissue (32). In addition, biological disease-modifying antirheumatic drugs (bDMARDs) targeting inflammatory cytokines have expanded the treatment options for patients with rheumatoid arthritis (RA) (33), inflammatory bowel disease, psoriatic arthritis, severe psoriasis, autoinflammatory disease, Castleman disease, and plaque psoriasis (**Table S1**) (34). As shown in **Table 1**, the therapies of tumor necrosis factor-α (TNF-α) targeting monoclonal antibody (Mab) adalimumab and IL-6 receptor (IL-6R) targeting Mab sarilumab could lead to injection site reactions (sarilumab), worsening RA (adalimumab) and increased incidences of infections (sarilumab: 28.8%; adalimumab: 27.7%) in patients (35). Moreover, it was reported that paradoxical inflammations such as psoriasiform lesions, arthritis are induced by anti-TNF Mabs in some patients with Crohn’s disease and ulcerative colitis (36). Furthermore, it has been found that anti-IL-1β Mab Canakinumab is associated with a higher incidence of fatal infection than placebo (30). Finally, it was reported that unfavorable responses on anti-IL-17A Mab secukinumab are driven by patients with elevated inflammatory markers such as C-reactive protein (37).

**Table 1:** Proinflammatory cytokine-blocking therapies paradoxically lead to increased inflammation.

Proinflammatory cytokines		Related findings	Changes of inflammation related genes	The related pathway	PMID
TNF	Human	The incidences of infections and worsening RA were about 27.7% and 0.5% in Adalimumab treatment.	N/A	N/A	27856432
		The risk of serious infections increased 2 folds in patients with RA treated with anti-TNF antibody.	N/A	N/A	16705109
		Paradoxical inflammation occurred involving the skin, joints and lungs under anti-TNF treatment in patients with inflammatory bowel disease.	IFN-α production; IL12B and IL23R increased.	type I IFN signaling; the differentiation of naïve T cells towards TH1 (via IL-12) or TH17 (via IL-23) cells	22751454
		The incidences of pneumonia were 2.2% and 1.4% using TNF antibodies Infliximab and Etanercept for RA.	N/A	N/A	20877307
		Anti-TNFα therapy up-regulated IL6 and IL23p19, in patients with Crohn’s disease; IL-1B and IL17A remained up-regulated in patients refractory to anti-TNF α.	IL-1B and IL17A are up-regulated in nonresponders.	IL17A pathway	24700437
		During anti-TNF therapy, there are upregulations of IL-23p19, IL23R, and IL-17A in patients with Crohn’s disease	IL23R, IL17A, IL17F and TNFR2 are up-regulated in nonrespinders.	IL23R signalling	29848778
	Mouse	TNF overexpression was cardioprotective	N/A	canonical NF-κB pathway signaling	26280121
		In macrophages, TNF produced less cytokines after challenged with LPS.	suppress IL6, TNF, IL-1β production	LPS-induced signaling	21602809
		In Tnf KO tumor tissues, tumor-promoting cytokines induced	the expression levels of IL-1b, IL-6, CXCL1 and CXCL2 increased.	COX-2/PGE2, IL-1b, IL-6 and CXCL1/2 pathways	23975421
IL1B	Human	For atherosclerotic therapy, incidence rates of deaths attributed to infection or sepsis in Canakinumab groups were higher.	N/A	N/A	28845751
		Severe infections were more frequent in Canakinumab group in patients with JIA.	N/A	N/A	23252526
IL6	Human	Incidences of infections were about 28.8% when Sarilumab monotherapy treat patients with RA.	N/A	N/A	27856432
		In multiple myeloma patients, anti-IL6 antibodies did not prevent IL6 production.	IL6	N/A	8823310
		Treating patients with RA with Tocilizumab increased infections.	N/A	N/A	21884601
	Mouse	IL-6 provided protection against influenza A infection.	Mcl-1 and Bcl-X L were down-regulated.	IL-6 or IL-6R signals	22294047
IL17A	Human	In patients with Crohn’s disease for treatment with Secukinumab, 51.3% infections were observed	CRP, and/or faecal calprotectin elevated	N/A	22595313
		Incidences of severe infection were 1% in Ixekizumab in the treatment of AS or RAS.	N/A	N/A	30360964
IL18	Human	Inhibition of IL-18 using GSK1070806 did not improve glucose control	N/A	N/A	26930607
	Mouse	Decrease in IL-18 in mice that were deficient in NLRP6 inflammasome was involved in enhanced colitogenic microbiota	NLRP6, ASC, caspase-1	NLRP6 flammasome pathway	21565393
		Il18 or Il18 receptor KO mice led to hyperphagia, obesity and insulin resistance	activation of STAT3 phosphorylation	STAT3 pathway	16732281

TNF, tumor necrosis factor; LPS, Lipopolysaccharide; IL, Interleukin; IFN, interferon; NF-κB, nuclear factor kappa B; CXCL, chemokine (C-X-C motif) ligand; COX-2/PGE2, prostaglandin-endoperoxide 2; Mcl-1, MCL1 apoptosis regulator; CRP, C-reactive protein; NLRP6, NLR family pyrin domain containing 6; ASC, apoptosis-associated speck-like protein; STAT3, signal transducer and activator of transcription 3; RA, Rheumatoid arthritis; JIA, juvenile idiopathic arthritis; AS, ankylosing spondylitis; RAS, radiographic axial spondyloarthritis; N/A, Not applicable.

The increased incidences of infections occurred when antibodies blocking proinflammatory cytokines were used to treat patients with inflammatory diseases. Experimental animal studies showed proinflammatory cytokine knockout or blocking can induce other cytokines production and activate some inflammation related pathways.

In addition, various inflammation paradoxes have been reported including new inflammations occur when: *i)* particular cytokine genes and inflammatory regulators are mutated (38); *ii)* patients experience somatic mutations (39) and inflammageing (40); *iii)* PCs are weakened due to single nucleotide polymorphism (41); *iv)* cytokine blockage therapies are used; *v)* genes encoded PCs and other regulators are knocked-out in mice; *vi)* inflammation is resurged when MHO undergoes a transition to classical metabolically unhealthy obesity (31, 32) in response to the long term stimulation of metabolic disease risk factors such as hyperlipidemia, danger associated molecular patterns (DAMPs) and conditional DAMPs as we reported (42); *vii)* an obesity paradox exists, wherein obese individuals survive sepsis at higher rates than their normal-weight counterparts (43); *viii)* inflammation paradoxes are observed in the Amazon region showing that the indigenous Tsimane in Bolivia appears protected against non-communicable metabolic inflammatory diseases (NCDs) such as obesity, type 2 diabetes, and CVDs despite increased inflammatory markers (44); and *ix)* A widely discussed physiological puzzle of mammalian pregnancy is the immunological paradox, the semi-allogenic fetus is not attacked by the mother’s adaptive immune system (45). These inflammation paradoxes were summarized in **Table S2**. In an attempt to solve these paradoxes, we examined a significant issue that remains unknown: why proinflammatory regulator blockage therapies lead to a “secondary wave of inflammation” (32).

Similar to single cytokine targeting Mab therapies discussed above, one of the current research strategies is to use gene-deficient mouse models and transgenic mouse models to determine the dominant effects of these inflammatory regulators in disease models such as atherogenesis (29, 46). Numerous disease risk factors have been identified to induce metabolic CVDs and other inflammatory diseases, such as hyperlipidemia, hyperglycemia, hyperhomocysteinemia, obesity, hypertension, and cigarette smoke (29, 47). It has been documented that inflammation and metaflammation are evolutionally conserved; the underlying pathways are cross-talking (48). However, the issue remains unknown whether in proinflammatory “regulator A” deficiency conditions, disease risk factors promote the regulator A-suppressed secondary wave of inflammation as we termed in our recent report (32).

InnateDB (https://www.innatedb.com/) is a comprehensive database on innate immune regulatory genes, which has been developed to facilitate systems-level investigations of the mammalian (human, mouse and bovine) innate immune response (49). This list of innate immune regulators in the InnateDB database (innatome) is especially useful for us to analyze the expression changes of innate immune regulators in the presence and absence of certain immune regulators. In order to improve our understanding on the expression changes in the innatome in the presence and absence of proinflammatory regulators, we examined our novel hypothesis that proinflammatory cytokine blockages induce inflammatory regulators. To test this hypothesis, we performed an extensive –omics database mining, determined the expressions of 1367 innate immune regulators (innatomic genes, IGs) in 18 microarrays after deficiencies of 15 PCs and made a set of significant findings. Our findings will provide novel insights on various inflammation paradoxes and PC regulation of IGs; and may re-shape new therapeutic strategies for various inflammations.

## Materials and Methods

### Expression Profile of IGs in Microarray Data For Patients With Various Inflammatory Diseases and Receiving Cytokine Targeting Mab Therapy for Various Proinflammatory Cytokine Gene Deficiencies

The 18 murine microarray datasets of proinflammatory cytokine gene deficiencies and eight microarray datasets of patients receiving Mab therapies were collected from National Institutes of Health (NIH)-National Center for Biotechnology Information (NCBI)-Gene Expression Omnibus (GEO) databases (https://www.ncbi.nlm.nih.gov/gds/) and analyzed with an online software GEO2R (https://www.ncbi.nlm.nih.gov/geo/geo2r/). The detailed information of these GEO datasets was shown in **Table 2A** and **2B** and related mechanism tables or figures. The original microarray experiments used different cells, which prevented us from comparing the effects of proinflammatory regulators in regulating IGs in the same cell types. Of note, our approach was well justified. For example, as a common practice, we (50) and others (51) often studied gene expression in non-ideal heterogenous peripheral blood mononuclear cell populations (PBMCs) in pathophysiological conditions, which are actually composed of many cell types (also see the Discussion section). The used IGs and ROS regulator gene -lists were listed in **Table S3**.

**Table 2A:** 18 microarray datasets were collected to analyze the changes of innate immunity molecules (innatomic genes, IGs) in deficiencies of proinflammatory regulators (*p* < 0.05, ∣log2FC∣>1).

No.	Factors	GEO NO.	Method	Innatomic genes (total n=1376)				Background	Cell type/tissue	PMID
				Up-regulatedN%		Down-regulatedN%				
	**Proinflammatory cytokines**						
1	TNF	GSE43145	Tnfa KO^a^	**52**	**3.78**	**24**	**1.74**	C57BL/6	Glandular stomach	23975421
2		GSE33253	Tnfr1,2 KO^b^	32	2.33	171	12.43	C57BL/6	Tumor endothelial	23056240
3	IFNG	GSE9892	Ifng KO^c^	81	5.89	138	10.03	BALB/c	Liver	19490417
4		GSE39592	Ifngr1 KO	**111**	**8.07**	**82**	**5.96**	C57BL/6	CD4+ T	23575689
5	IL1B	GSE15750	Traf6 KO**^#^**	10	0.73	22	1.60	C57BL/6	CD8 T	19494812
6		GSE73875	Irak1 KO	**7**	**0.51**	**0**	**0.00**	C57BL/6	CD4+ CD26L+ T	26561545
7	IL6	GSE63761	Il6 KO	**44**	**3.20**	**13**	**0.94**	C57BL/6	adipose	25738456
8	IL17	GSE88800	Il17ra KO	**141**	**10.25**	**50**	**3.63**	N/A	kidney	27814401
9	IL18	GSE64308	Il18 KO	**48**	**3.49**	**12**	**0.87**	C57BL/6	brown adipose	30453990
10		GSE64309	Il18 KO	40	2.91	51	3.71	C57BL/6	Liver	27063959
11		GSE64310	Il18 KO	**10**	**0.73**	**6**	**0.44**	C57BL/6	Kidney	29514661
	**ProInflammatory related transcription factor**									
12	STAT	GSE40666	Stat1 KO	**48**	**3.49**	**25**	**1.82**	C57BL/6	CD8 T	22968462
13		GSE6846	Stat3 KO	**108**	**7.85**	**32**	**2.33**	N/A	pulmonary type II epithelia	18070348
14	NFKB	GSE45755	Rela KO	**26**	**1.89**	**3**	**0.22**	C57BL/6	lineage-Flk2-c-kit+Sca-1+	23670180
15		GSE30049	Ikk2 KO	47	3.42	83	6.03	N/A	tumor-derived cell line	22327365
	**Inflammatory-related miRNAs**									
16	MIR155	GSE45122	mir155 KO	**21**	**1.53**	**13**	**0.94**	C57BL/6	CD4+ IL-17F RFP+ T	23686497
17		GSE66815	mir155 KO	**86**	**6.25**	**82**	**5.96**	C57BL/6	spleen	25911753
18	MIR221	GSE19777	MIR221 KD	18	1.31	39	2.83	N/A*	breast cancer	21057537

Up, up-regulated IGs; Down, down-regulated IGs; KO, Knockout; KD, Knockdown; N/A, Not applicable; The significant differential expressed IGs were the comparison results between the major inflammatory KO and the parallel control. Please note: ^a^Tnfa KO Gan mice vs. WT Gan mice; ^b^Tnfr1,2 KO B16F1 melanoma tumors vs. WT B16F1 melanoma tumors; ^c^based on the Tgfb KO model mice of autoimmune hepatitis; #the combined data of Traf6 KO 6 days and 10 days because of the small number of the regulated innate immune genes; *Homo sapiens.

Tnfr, tumor necrosis factor receptor superfamily; Ifngr1, interferon gamma receptor 1; Traf6, TNF receptor associated factor 6. It mediates signaling from members of the TNF receptor superfamily as well as the Toll/IL-1 family; Irak1, interleukin-1 receptor-associated kinase 1; Rela, v-rel reticuloendotheliosis viral oncogene homolog A; Ikk2, inhibitor of kappaB kinase beta.

In gene KO experiments, for the pro-inflammatory cytokines, we focus on the up-regulated innate immune genes, those maybe inhibited by the proinflammatory cytokines during inflammation.

Table showing the number and the ratio of the up-regulated and down-regulated IGs in proinflammatory molecules KO or KD microarrays. From the data, in majority microarrays, the ratio of up-regulated IGs is higher than that of down-regulated IGs (marked in bold). **Table S4** listed the housekeeping genes changes, the criteria for selecting a database in this study based on the housekeeping genes changes with p > 0.05 or∣log2FC∣<1 between treated group and control group. **Table S5** listed the detailed expression changes of IGs.

**Table 2B:** The innatomic genes (IGs) were analyzed in cytokine-monoclonal antibodies therapy microarrays.

GEO#	Disease	Target	Drug	Tissue	Comparation	IGs		PMID
						up	down	
GSE15602	rheumatoid arthritis	TNF	Adalimumab	synovium	poor responder vs. good responder	**73**	**67**	19389237
GSE111761	Crohn’s disease	TNF	Infliximab or Adalimumab	intestine	none-responder vs. responder	**381**	**113**	29848778
GSE92415	ulcerative colitis	TNF	Golimumab	colonic mucosa	none-responder vs. responder (6 weeks)	**18**	**2**	29981298
GSE14580	ulcerative colitis	TNF	Infliximab	colonic mucosa	none-responder vs. responder	**106**	**14**	19700435
GSE24742	rheumatoid arthritis	TNF	Rituximab	synovium	good-responder 12 weeks vs.0 week	29	42	21337318
	rheumatoid arthritis	TNF	Rituximab	synovium	moderate-responder 12 weeks vs.0 week	21	73	21337318
	rheumatoid arthritis	TNF	Rituximab	synovium	poor-responder 12 weeks vs.0 week	**33**	**28**	21337318
GSE45867	rheumatoid arthrtis	IL6R	Tocilizumab	synovium	after therapy 12 weeks vs.before	2	23	24449571
GSE31652	psoriasis vulgaris	IL17A	LY2439821	skin	LY2439821 2 weeks vs.0 weeks	47	87	22677045

The results showed that the IGs were more down-regulated than up-regulated after therapy compared with before therapy or with placebo; the IGs were more up-regulated than down-regulated when poor responder or non-responder compared with responder or good responder (marked in bold). **Table S6** listed the detail expression changes of IGs in cytokine-monoclonal antibodies therapy microarrays.

### Statistical Analysis of Microarray Data

Six house-keeping genes including ACTB, CHMP2A, RPL27, SRP14, RPL22 and OAZ1 (**Table S4**) in all GEO datasets regardless of species that were chosen for this study. The house-keeping gene list was extracted from the list provided by Eisenberg and de Jonge (52, 53). Briefly, the mean fold change (FC) of house-keeping genes between treatment and control groups vary from 0.79 to 1.13 (53). As this variation was very narrow, we concluded that the datasets (**Table 2A** and **2B**) were of high quality. Genes with expression changes more than 2-folds in microarrays were defined as the upregulated genes, while genes whose expressions decreased more than 2-fold in microarrays were defined as downregulated genes. Simply, genes with the expression changes at |log2FC|>1 and *p*<0.05 were defined as the differentially expressed genes.

### Ingenuity Pathway Analysis

We utilized Ingenuity Pathway Analysis (IPA, Qiagen, https://www.qiagenbioinformatics.com/products/ingenuity-pathway-analysis/) to characterize clinical relevance and molecular and cellular functions related to the identified genes in our microarray analysis. Differentially expressed genes were identified and uploaded into IPA for analysis. The core and pathways analyses were used to identify molecular and cellular pathways, as we have previously reported (54, 55).

## Results

### Proinflammatory Cytokines (PCs) Suppress Innatomic Genes (IGs); Upregulated IGs in the Deficiencies of IFNγ, IFNγR1, IL-17A, STAT3, and miR155 Are More Than That of the Deficiencies of TNFα, IL-1β, IL-6, IL-18, STAT1, NF-kB, and miR221

**Figure 1:**
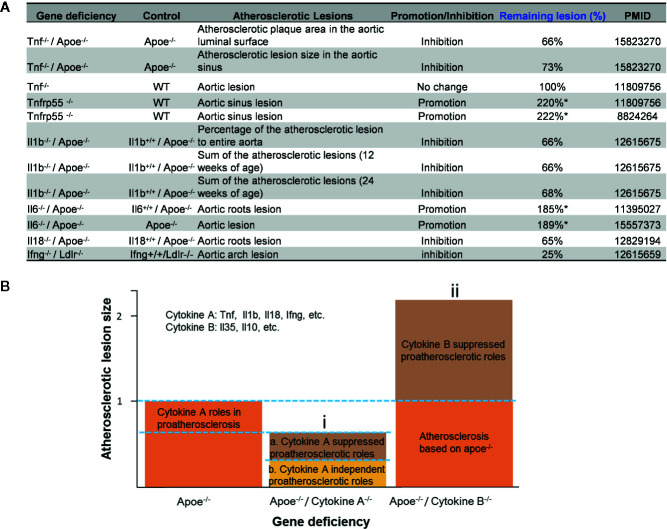
Proinflammatory cytokine-suppressed proinflammatory/proatherogenic mechanisms contribute to atherosclerotic lesions remained in mouse models with cytokine deficiencies. **(A)** Cytokine deficiency can only inhibit smaller atherosclerotic lesions even promote (Remaining lesion size > 100%) atherosclerotic lesions in murine models of atherosclerosis. **(B)** Cytokine roles were divided into two major groups: (i) Cytokines such as Tnf, Il1b, Il18 and Ifng were identified as cytokines with pro- atherosclerotic roles; and (ii) cytokines such as IL-35 and IL-10 (not focused in this study) were identified as cytokines with anti-atherosclerotic roles. In order to better compare the lesions size changes after cytokine deficiency, atherosclerotic lesions size is defined “100% (1)” in murine models of atherosclerosis (Apoe-/- mouse).

The studies using the compound gene-deficient mice established with specific cytokine deficiency crossing to two atherogenic mouse models such as apolipoprotein E deficient (ApoE^-/-^), and low-density lipoprotein receptor-deficient (LDLR^-/-^) background have significantly improved our understanding on the roles of these PCs on atherosclerotic progression. Deficiencies of TNFα, IL-1β, IL-18 and interferon-γ (IFNγ) lead to a significant reduction of atherosclerotic lesions. However, the parts of atherosclerotic lesions remain in those proinflammatory cytokine deficient and ApoE^-/-^ double gene KO mice. The atherosclerotic lesions remain 66% for TNF^-/-^/ApoE^-/-^, 66% for IL-1β^-/-^/ApoE^-/-^, 65% for IL-18^-/-^/ApoE^-/-^ and 25% for IFNγ^-/-^/LDLR^-/-^ mice, respectively (**Figure 1A** **)**. The remained atherosclerotic lesions may be contributed towards by the following factors (**Figure 1B**): *1)* the specific cytokine “A”-independent proinflammatory, proatherogenic cytokines and factors, and *2)* proinflammatory, proatherogenic cytokines and factors suppressed by the specific cytokine “A”. These two factors, especially the proinflammatory/proatherogenic cytokines/factors suppressed by the specific deficient cytokine “A”, remained unknown. In addition, we proposed a recently so-called “second wave of inflammatory responses” (32) for proinflammatory miR155 suppressed proinflammatory adipokines leptin and resistin, which may result from direct suppression of these proinflammatory adipokines by miR155 or indirect suppression of adipogenesis by miR155 (31). The second wave of inflammatory responses is required to protect the organism from infections or other pathologies at least when the following situations occur in the paradoxes introduced above when PCs are deficient and/or downregulated. Moreover, as shown in **Table 1**, proinflammatory cytokine-blocking therapies may paradoxically lead to increased inflammation, and increased incidences of infections. Finally, experimental animal studies (**Table 1**), with gene-deficient mouse models of PCs, proinflammatory cytokine knockout or blocking induced other cytokines production and activate some inflammation-related pathways was shown. In summary, our and others’ reports demonstrated that in some diseases and pathophysiological relevant conditions when proinflammatory regulators are deficient or inhibited, other PCs and IGs are upregulated.

Since the pathways underlying inflammations and **meta**flammations are cross-talked (48), the interaction modes can be classified into three categories: *i)* agonism and synergy; *ii)* inhibition and antagonism; and *iii)* parallel and independence. To determine the mechanisms underlying cytokine targeting Mab therapies-induced inflammation and PC deficiencies-induced inflammation, we hypothesize that the deficiencies of PCs upregulate IGs in addition to downregulating IGs. We applied the -omics database mining methods and principles that we pioneered in 2004 (56, 57) to this study. The detailed features and justification of this data mining approach were highlighted in **Table 1** of our recent paper (47). We examined a comprehensive list of IGs collected in the InnateDB database (https://www.innatedb.com/), which had a total of 1367 genes. Panoramic profiling of the expressions of IGs in PC deficiency microarray datasets would lead to high throughput characterization on transcriptomic regulation of the PCs on IGs. **Table 2A** and **2B** are the results of the changes of IGs in deficiencies of PCs and in cytokine-monoclonal antibodies therapy microarrays, respectively. **Table S5** and **Table S6** listed the detailed expression changes of IGs. As shown in **Table 2A**, we found 11 cytokine gene knock-out (KO) microarray datasets, four proinflammatory transcription factor KO datasets, three proinflammatory microRNA KO datasets, including TNFα and TNFR1,2 KO, IFNγ and IFNγ receptor 1 (IFNγR1) KO, IL-1β pathway-related TNF receptor associated factor 6 (TRAF6) and interleukin-1 receptor-associated kinase 1 (IRAK1) KO, one IL-6 KO, one IL-17 receptor A (IL-17RA) KO, three IL-18 KO, one signal transducer and activator of transcription protein 1 (STAT1) KO, one STAT3 KO, one NF-kB subunit Rela KO, one Inhibitor of NFKB kinase subunit-β (IKK2) KO, two microRNA-155 (miR155) KO and one miR221 KD (knock-down). The results in **Table 2A** showed that: *i)* the deficiencies of all the 18 proinflammatory regulators lead to upregulation of IGs from 0.51% to 10.25% out of a total of 1367 IGs; *ii)* upregulated IGs in the deficiencies of IFNγ, IFNγR1, IL-17A, STAT3 and miR155 are more than that of the deficiencies of TNFα, IL-1β, IL-6, IL-18, STAT1, NF-kB, and miR221; and *iii)* the deficiencies of IFNγ, IFNγR1, IL-17RA, STAT3 and miR155 lead to high upregulation of IGs by 5.89%, 8.07%, 10.25%, 7.85% and 6.25%, respectively, in normal cell types and tissues including CD4^+^ T cells (IFNγ KO), kidney (IL-17 KO), pulmonary type II epithelial cells (STAT3 KO) and spleen (miR155 KO). Of note, the roles of IL-17 in promoting atherosclerosis development have been controversial as we (58), and others reported (46). However, the pro-atherogenic roles of TNFα and IFNγ have been well documented (46, 59). Therefore, our results have demonstrated for the first time that PCs suppress the expressions of some IGs; and upregulated IGs in the deficiencies of IFNγ, IFNγR1, IL-17RA, STAT3 and miR155 are more than that in the deficiencies of TNFα, IL-1β, IL-6, IL-18, STAT1, NF-kB, and miR221. These results have suggested that IFNγ, IFNγR1, IL-17RA, STAT3 and miR155 suppress more IGs expressions than other cytokines.

In microarray datasets of patients receiving Mab therapy (**Table 2B**), the results showed that more IGs were downregulated than upregulated when after therapy compared with the group before therapy or with placebo; more IGs were upregulated than downregulated when poor responder or non-responder compared with the group responders or good responders. On the one hand, the results confirm the efficacy of Mab in most of patients with autoimmune diseases, on the other hand, the results cannot deny the condition that in drug none-responders: “suppressed cytokines or innate immune regulator molecules” were upregulated.

**Figure 2:**
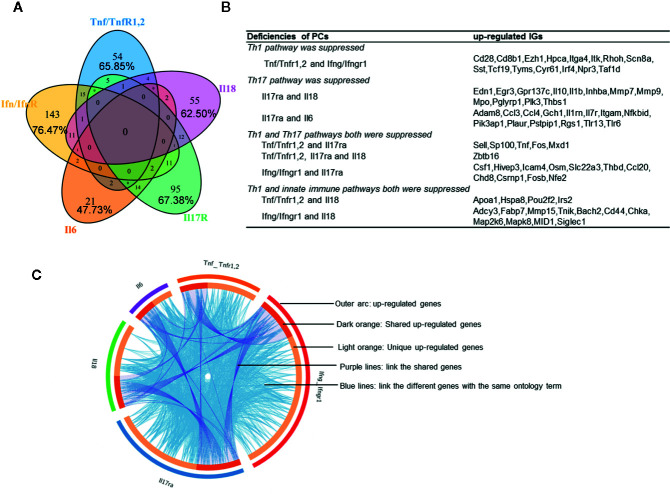
Deficiencies of proinflammatory cytokines shared the up-regulated innatomic genes. **(A)** 82, 187, 44, 141, and 88 up-regulated non-repeating innatomic genes were found in Tnf/Tnfr1,2 knock-out (KO), Ifn/Ifnr1 KO, Il6 KO, Il17ra KO and Il18 KO five groups according to the **Table 2A**, respectively. Venn Diagram (generated by using R) showed the majority of proinflammatory cytokine-suppressed innatomic genes were cytokine-specific and accounted for 65.85%, 76.47%, 47.73%, 67.38%, 62.50% in Tnf/Tnfr1,2 KO, Ifn/Ifnr1 KO, Il6 KO, Il17ra KO and Il18 KO groups, respectively. **(B)** There were several common genes between two or among three groups. In state of Th1 pathway was suppressed: in Tnf/Tnfr1,2 and Ifn/Ifnr1 KO groups, Cd28, isotype switching positive regulating gene, Cd8b1, activate T cell, Itga4, positively regulate leukocyte tethering or rolling, Itk, NK T cell differentiation and phospholipase C activity, and T-helper 17 cell lineage commitment and differentiation gene (Irf4) were commonly up-regulated. In state of Th17 pathway was suppressed: in Il17ra and Il18 KO groups, Egr3, has the role of regulating gamma-delta T cell differentiation and Il1b, positively regulate T-helper 1 cell cytokine production were up-regulated; in Il17ra and Il6 KO groups, Ccl3, which has the role of release of sequestered calcium ion into cytosol by sarcoplasmic reticulum and Ccl4, positively regulate natural killer cell chemotaxis, Il7r, positively regulate T cell differentiation in thymus, Nfkbid, positively regulate T-helper 17 cell differentiation, and Pik3ap1, related to toll-like receptor 2,7,9 related signal pathways were up-regulated. In state of Th1 and Th17 pathways both were suppressed: in Tnf/Tnfr1,2, Il17ra KO groups, Sell, related to leukocyte tethering or rolling, neutrophil degranulation and leukocyte adhesion to vascular endothelial cell, was common up-regulated; in Tnf/TnfR1,2, Il17ra and Il18 KO groups, Zbtb16, which positively regulate NK T cell differentiation was up-regulated; in Ifn/Ifnr1 and Il17ra KO groups, Osm, positive regulation of interleukin-17 secretion, Thbd, negative regulation of platelet activation, and Ccl20, positive regulation of thymocyte, T cell and lymphocyte migration, were up-regulated. In state of Th1 and innate immune pathways both were suppressed: in Tnf/Tnfr1,2, and Il18 KO groups, Irs2, which has the role of negative regulation of plasma membrane long-chain fatty acid transport was up-regulated; in Ifn/Ifnr and Il18 KO groups, Cd44, which has the role of positive regulation of monocyte aggregation, was up-regulated. **(C)** The Circos plot (generated by using Metascape http://metascape.org/gp/index.html#/main/step1) showed how genes overlap from the up-regulated genes in these five groups, including the same genes (purple lines link the same genes that are shared by multiple proinflammatory cytokines KO) and genes with the same ontology term (blue lines link the different genes where they fall into the same ontology term) shared by the five groups.

We hypothesized that PCs are cross-talked and share their regulation on the expression of IGs. To test this hypothesis, we performed a Venn Diagram analysis on the upregulated IGs from proinflammatory cytokine KO datasets. Among 15 IGs lists shared by two proinflammatory cytokine pathways, the five shared cytokine pathways including TNFα/IFNγ, IFNγ/IL-18, IL-6/IL-17, IFNγ/IL-17, and IL-17/IL6 pathways have the higher numbers of shared IGs (**Figures 2A, B**). We further examined the cross-talking among all the five cytokine pathways using the Circos Plot (http://metascape.org/gp/index.html#/main/step1) (**Figure 2C**), Comparing with the data analyzed with the Venn Diagram, the Circos plot analysis has advantages in including the different genes fall into the same ontology term (Blue lines) among the IGs upregulated in the deficiencies of five PCs. The outer circle showed the upregulated IGs in each cytokine pathway. The dark orange section in the inner circle indicated the shared IGs, and the light orange bar indicated the cytokine pathway unique IGs. The results showed that: *1)* each cytokine deficiency-upregulated IGs were shared with other cytokine pathways; *2)* the dark orange section in the inner circle indicated the shared IGs (Purple lines link the same gene), which were the same as the cytokine-shared IGs in **Figure 2A**; and *3)* IFNγ/IFNγR1 pathways have the highest numbers of upregulated IGs, which had minimal shared IGs with that of IL-6 pathway and had more shared IGs with that of IL-17, TNFα and IL18. Our findings were correlated well with that reported: IFNγ is an essential cytokine expressed highly in type 1 T helper cells (Th1); and IL-17/IFNγ double producing cells, the so-called Th17/Th1 plastic subset (21, 60), is a phenotype frequently observed in pathological conditions (61). These results have demonstrated that the deficiencies of PCs upregulate IGs, which can be in both cytokine-specific and shared manners.

### IFNγR1 and IL-17 Inhibit 59 and 39 Pathways, Respectively; in Contrast, TNFα, IL-6 and IL-18 Inhibit Only Four to Five Pathways

We then examined the signaling pathways in upregulated IGs in the deficiencies of five PCs such as TNFα, IFNγ, IL-6, IL-17 and IL-18. We used IPA to perform this analysis, which is a web-based bioinformatics application that allows the uploading of microarray and RNA-Seq data for functional pathway analysis and integration. **Table 3A** and **3B** are the IPA results of upregulated and downregulated IGs in deficiencies of PCs. The detailed IPA results were showed in **Table S7** and **Table S8**. As shown in **Table 3A**, among 86 significant pathways identified, four pathways such as neuroinflammation, cardiac hypertrophy, leukocyte extravasation, and colorectal cancer metastasis were shared by four cytokine pathway deficiencies; 12 pathways including an additional eight pathways such as dendritic cell maturation, B cell receptor signaling, Tec kinase, nitric oxide and reactive oxygen species in macrophages, integrin, FGF signaling, ILK signaling and PI3 kinase in B cells, were shared by three cytokine pathway deficiencies, 28 pathways were shared by two cytokine pathways; the rest of the 58 pathways were induced by single cytokine deficiencies. In addition, the deficiencies by IFNγR1 KO and IL17R KO upregulate 59 and 39 pathways, respectively. The deficiencies of IFNγR1 and IL17RA resulted in the upregulation of many unique signaling pathways, suggesting that IFNγ and IL-17 inhibit many proinflammatory pathways. However, PD-1, PD-L1 cancer immunotherapy pathway was downregulated in IFNγ pathway deficiency, suggesting that IFNγ is required for PD-1, PD-L1 cancer immunotherapy presumably *via* expanding type 1 CD4^+^ T helper cell (Th1) pathways. Moreover, the deficiencies of TNFα, IL-6 and IL-18 resulted in fewer pathways upregulated, including four TNFα suppressed pathways (dendritic cell maturation, Tec kinase signaling, oxygen species in macrophages, and interferon signaling), five IL6 suppressed pathways (neuroinflammation signaling, leukocyte extravasation, colorectal cancer metastasis, B cell receptor signaling, and PI3K signaling in B cells), and five IL-18 suppressed pathways (neuroinflammation signaling, cardiac hypertrophy, NF-kB signaling, acute phase response signaling, endocannabinoid cancer initiation).

**Table 3A:** Ingenuity Pathway Analysis (IPA) results showed the significant pathways (∣Z score∣> 2) of up-regulated innatomic genes (IGs) in proinflammatory cytokine KO microarray datasets.

No.	Significant signaling pathways	Tnf-/- (GSE43145)	Tnfr1,2-/- (GSE33253)	Ifng-/- (GSE9892)	Ifngr1-/- (GSE39592)	Il6-/- (GSE63761)	Il17ra-/- (GSE88800)	Il18-/- (GSE64308)
**1**	**Neuroinflammation Signaling Pathway**				↑	↑	↑	↑
2	Cardiac Hypertrophy Signaling (Enhanced)			↑	↑		↑	↑
**3**	**Leukocyte Extravasation Signaling**			↑	↑	↑	↑	
4	Colorectal Cancer Metastasis Signaling			↑	↑	↑	↑	
**5**	**Dendritic Cell Maturation**	↑			↑		↑	
6	B Cell Receptor Signaling				↑	↑	↑	
7	Tec Kinase Signaling	↑			↑		↑	
**8**	**Production of Nitric Oxide and Reactive Oxygen Species in Macrophages**	↑			↑		↑	
9	Integrin Signaling			↑	↑		↑	
10	FGF Signaling			↑	↑			
11	ILK Signaling			↑	↑		↑	
**12**	**PI3K Signaling in B Lymphocytes**				↑	↑	↑	
**13**	**PD-1, PD-L1 cancer immunotherapy pathway**			↓	↓			
**14**	**NF-κB Signaling**				↑			↑
15	Role of NFAT in Regulation of the Immune Response			↑	↑			
16	Acute Phase Response Signaling						↑	↑
**17**	**HMGB1 Signaling**				↑		↑	
18	RANK Signaling in Osteoclasts				↑		↑	
19	Type II Diabetes Mellitus Signaling				↑		↑	
20	Adrenomedullin signaling pathway				↑		↑	
21	LPS-stimulated MAPK Signaling				↑		↑	
**22**	**NF-κB Activation by Viruses**				↑		↑	
23	Signaling by Rho Family GTPases				↑		↑	
24	Cholecystokinin/Gastrin-mediated Signaling				↑		↑	
**25**	**IL-8 Signaling**				↑		↑	
**26**	**PKCθ Signaling in T Lymphocytes**				↑		↑	
**27**	**Toll-like Receptor Signaling**				↑		↑	
28	IL-1 Signaling				↑		↑	
**29**	**Interferon Signaling**	↑						
30	ERK/MAPK Signaling		↑					
31	Mouse Embryonic Stem Cell Pluripotency		↑					
**32**	**Fcγ Receptor-mediated Phagocytosis in Macrophages and Monocytes**			↑				
**33**	**Th2 Pathway**			↑				
**34**	**Systemic Lupus Erythematosus In T Cell Signaling Pathway**			↑				
35	Endocannabinoid Cancer Inhibition Pathway							↑
36	NGF Signaling				↑			
37	Cardiac Hypertrophy Signaling				↑			
38	Synaptogenesis Signaling Pathway				↑			
39	Fc Epsilon RI Signaling				↑			
**40**	**CD28 Signaling in T Helper Cells**				↑			
41	EGF Signaling				↑			
42	Rac Signaling				↑			
43	PDGF Signaling				↑			
**44**	**iCOS-iCOSL Signaling in T Helper Cells**				↑			
45	Pancreatic Adenocarcinoma Signaling				↑			
46	Ephrin Receptor Signaling				↑			
47	Cdc42 Signaling				↑			
48	p70S6K Signaling				↑			
49	Phospholipase C Signaling				↑			
50	Neurotrophin/TRK Signaling				↑			
51	ErbB Signaling				↑			
**52**	**CD27 Signaling in Lymphocytes**				↑			
53	Neuregulin Signaling				↑			
54	Endocannabinoid Developing Neuron Pathway				↑			
55	PFKFB4 Signaling Pathway				↑			
56	GNRH Signaling				↑			
57	Glioma Signaling				↑			
58	Melatonin Signaling				↑			
59	Role of NFAT in Cardiac Hypertrophy				↑			
60	Acute Myeloid Leukemia Signaling				↑			
61	Opioid Signaling Pathway				↑			
62	Sphingosine-1-phosphate Signaling				↑			
63	G Beta Gamma Signaling				↑			
64	Gα12/13 Signaling				↑			
65	Gαq Signaling				↑			
66	Wnt/β-catenin Signaling				↑			
67	Thrombin Signaling				↑			
**68**	**TREM1 Signaling**						↑	
**69**	**IL-6 Signaling**						↑	
70	LXR/RXR Activation						↓	
**71**	**iNOS Signaling**						↑	
72	PPAR Signaling						↑	
**73**	**Systemic Lupus Erythematosus In B Cell Signaling Pathway**						↑	
74	Type I Diabetes Mellitus Signaling						↑	
75	p38 MAPK Signaling						↑	
76	Antioxidant Action of Vitamin C						↓	
77	MIF Regulation of Innate Immunity						↑	
78	Inflammasome pathway						↑	
79	Osteoarthritis Pathway						↑	
80	STAT3 Pathway						↑	
81	VDR/RXR Activation						↑	
82	Role of IL-17F in Allergic Inflammatory Airway Diseases						↑	
**83**	**FAT10 Cancer Signaling Pathway**						↑	
84	Angiopoietin Signaling						↓	
**85**	**T Cell Exhaustion Signaling Pathway**						↑	
86	Endothelin-1 Signaling						↑	

A total of 11 microarrays about proinflammatory cytokines in **Table 2A** were analyzed, GSE15750, GSE73875, GSE64309 and GSE64310 were not included because there were no significant (∣Z score∣< 2) pathways in them.

The data showed a total of 28 shared significant signaling pathways of the up-regulated innate immune genes in PCs KO microarrays. 12 cellular immune response signals were activated and PD-1, PD-L1 cancer immunotherapy pathway was suppressed by the up-regulated IGs (marked in bold). And in 58 unique significant signaling pathways, 12 cellular immune response signals (marked in bold) are activated when one proinflammatory cytokine was KO. The detailed IPA results were showed in **Table S7**.

**Table 3B:** IPA results showed the significant pathways (∣Z score∣> 2) of down-regulated IGs in pro-inflammatory cytokine KO microarray datasets.

No.	Significant signaling pathways	Tnfr1,2-/- (GSE33253)	Ifng-/- (GSE9892)	Ifngr1-/- (GSE39592)	Il18-/- (GSE64309)
**1**	**Dendritic Cell Maturation**	↓	↓	↓	↓
2	Cardiac Hypertrophy Signaling (Enhanced)	↓	↓	↓	
**3**	**Systemic Lupus Erythematosus In B Cell Signaling Pathway**	↓	↓	↓	
**4**	**Interferon Signaling**	↓	↓	↓	
**5**	**Role of Pattern Recognition Receptors in Recognition of Bacteria and Viruses**	↓	↓	↓	
**6**	**TREM1 Signaling**	↓	↓	↓	
**7**	**Neuroinflammation Signaling Pathway**	↓	↓	↓	
8	iNOS Signaling	↓	↓	↓	
**9**	**p38 MAPK Signaling**	↓	↓	↓	
**10**	**Production of Nitric Oxide and Reactive Oxygen Species in Macrophages**	↓	↓	↓	
11	Colorectal Cancer Metastasis Signaling	↓	↓	↓	
**12**	**PI3K Signaling in B Lymphocytes**	↓	↓	↓	
13	IL-1 Signaling	↓	↓	↓	
14	Adrenomedullin signaling pathway	↓	↓		↓
15	Cholecystokinin/Gastrin-mediated Signaling	↓	↓		↓
**16**	**Toll-like Receptor Signaling**		↓	↓	↓
17	HGF Signaling	↓	↓		
18	Tec Kinase Signaling	↓	↓		
**19**	**MIF Regulation of Innate Immunity**	↓	↓		
**20**	**NF-κB Activation by Viruses**	↓	↓		
21	Signaling by Rho Family GTPases	↓	↓		
22	ERK/MAPK Signaling	↓	↓		
**23**	**IL-8 Signaling**	↓	↓		
24	PI3K/AKT Signaling	↓	↓		
**25**	**PKCθ Signaling in T Lymphocytes**	↓	↓		
26	Endothelin-1 Signaling	↓	↓		
27	Synaptogenesis Signaling Pathway	↓	↓		
28	ILK Signaling	↓	↓		
29	PDGF Signaling	↓	↓		
**30**	**Activation of IRF by Cytosolic Pattern Recognition Receptors**	↓		↓	
**31**	**IL-6 Signaling**	↓		↓	
**32**	**Th17 Activation Pathway**	↓		↓	
33	JAK/Stat Signaling	↓		↓	
**34**	**NF-κB Signaling**			↓	↓
**35**	**HMGB1 Signaling**		↓	↓	
36	Cardiac Hypertrophy Signaling		↓	↓	
37	Acute Phase Response Signaling			↓	↓
38	Rac Signaling	↓			
39	B Cell Receptor Signaling	↓			
40	Ephrin Receptor Signaling	↓			
41	Role of IL-17F in Allergic Inflammatory Airway Diseases	↓			
42	PPAR Signaling	↑			
**43**	**Leukocyte Extravasation Signaling**	↓			
44	TNFR1 Signaling	↓			
**45**	**MIF-mediated Glucocorticoid Regulation**	↓			
46	Integrin Signaling	↓			
47	RhoA Signaling	↓			
48	Type II Diabetes Mellitus Signaling	↓			
49	Cdc42 Signaling	↓			
50	Antioxidant Action of Vitamin C	↑			
**51**	**iCOS-iCOSL Signaling in T Helper Cells**	↓			
**52**	**FAT10 Cancer Signaling Pathway**	↓			
53	PAK Signaling	↓			
54	Type I Diabetes Mellitus Signaling	↓			
55	Renin-Angiotensin Signaling	↓			
**56**	**CD28 Signaling in T Helper Cells**	↓			
**57**	**IL-7 Signaling Pathway**	↓			
58	LPS-stimulated MAPK Signaling	↓			
59	Fc Epsilon RI Signaling		↓		
60	cAMP-mediated signaling		↓		
61	Osteoarthritis Pathway		↓		
62	Hepatic Fibrosis Signaling Pathway		↓		
**63**	**T Cell Exhaustion Signaling Pathway**		↓		
64	Oncostatin M Signaling		↓		
65	Unfolded protein response		↓		
66	Retinoic acid Mediated Apoptosis Signaling		↓		
67	LXR/RXR Activation		↑		
**68**	**Role of NFAT in Regulation of the Immune Response**		↓		
69	PPARα/RXRα Activation		↑		
70	RhoGDI Signaling		↑		

A total of 10 microarrays about proinflammatory cytokines in **Supplementary Table 2A** were analyzed, GSE43145, GSE73875, GSE88800, GSE64309, GSE63761, GSE64308 and GSE64310 were not included because there were **no significant** (∣Z score∣< 2) pathways in them. The data showed a total of 37 shared significant signaling pathways of the down-regulated innate immune genes in the inflammatory cytokines KO microarrays. According to the IPA classification, 19 cellular immune response signals (marked in bold) are down-regulated by the PCs KO. And in 33 unique significant signaling pathways, 8 cellular immune response signals (marked in bold) were suppressed when one PCs was KO. The detailed IPA results were showed in **Table S8**.

We also examined the signaling pathways in downregulated IGs in the deficiencies of four PCs such as TNFαR, IFNγ, IFNγR1, and IL-18. As shown in **Table 3B**, among 70 significant pathways identified, the dendritic cell maturation pathway was downregulated in all four cytokine deficiencies investigated. Sixteen pathways such as cardiac hypertrophy signaling, systemic Lupus erythematosus in B cell signaling, etc. were downregulated in three cytokine deficiencies. Thirty-seven pathways were downregulated in two cytokine deficiencies. In addition, TNFαR deficiency resulted in the downregulation of 51 pathways. IFNγ deficiency led to the downregulation of 38 pathways, and IFNγR1 deficiency resulted in the downregulation of 22 pathways. IL-18 deficiency led to the downregulation of 6 pathways. Of note, the IPA is an experimental database with the focus on pathway analysis, similar to the NIH-NCBI-PubMed database, rather than bioinformatic prediction. What the IPA found that IGs inhibited by IFNγR1 and IL-17 are functionally involved in 59 and 39 pathways in a statistically significant manner, suggesting that inhibitions of IGs by those two cytokines are multiple pathways-, and multiple function-based. In comparison, the IPA found that inhibitions of IGs by TNF-α, IL-6 and IL-18 are four to five pathways-based, much more focused and specific than that inhibited by IFNγR1 and IL-17. These results have demonstrated that IFNγ/IFNγR1 and IL-17 inhibit 59 and 39 pathways, respectively, whereas the deficiencies of TNFα, IL-6 and IL-17 inhibit four to five out of 86 pathways.

### The IFNγ Promoted and -Suppressed Programs Have 4 Shared Pathways; IFNγR1-Promoted and -Suppressed Programs Have 11 Shared Pathways; and miR155-Promoted and -Suppressed Programs Have 13 Shared Pathways, Suggesting Negative-Feedback Conserved Mechanisms

**Figure 3:**
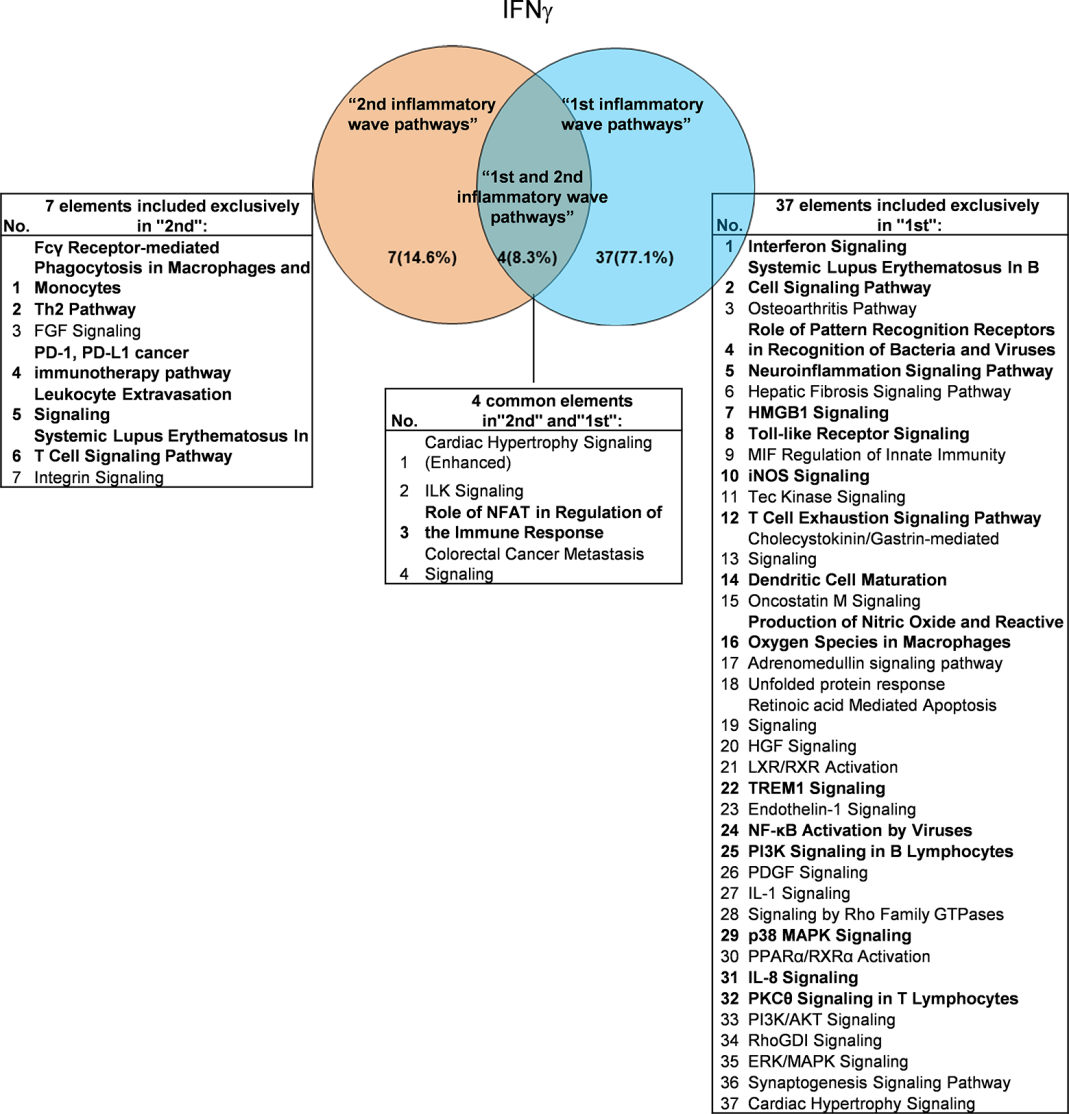
Venn diagram showing the overlapping significant pathways of up-regulated and down-regulated IGs in Ifng KO microarray dataset (GSE9892). The shared pathways by up-regulated and down-regulated IGs were called “2nd and 1st inflammatory wave pathways”. The data showed in 4 common significant “2nd and 1st inflammatory wave pathways”, Role of NFAT in Regulation of the Immune Response is immune response related pathway. The data suggested that the up-regulated IGs have the same immune function to the down-regulated IGs by Ifng KO. Fcγ Receptor-mediated Phagocytosis in Macrophages and Monocytes, Th2 Pathway, PD-1, PD-L1 cancer immunotherapy pathway, Leukocyte Extravasation Signaling and Systemic Lupus Erythematosus In T Cell Signaling Pathway were unique cellular immune response pathways of “2nd inflammatory wave pathways”. The suppression of Interferon Signaling, Systemic Lupus Erythematosus In B Cell Signaling Pathway, and Role of Pattern Recognition Receptors in Recognition of Bacteria and Viruses, etc. 16 pathways were unique cellular immune response pathways of “1st inflammatory molecules”. The cellular immune response pathways were marked in bold.

**Figure 4:**
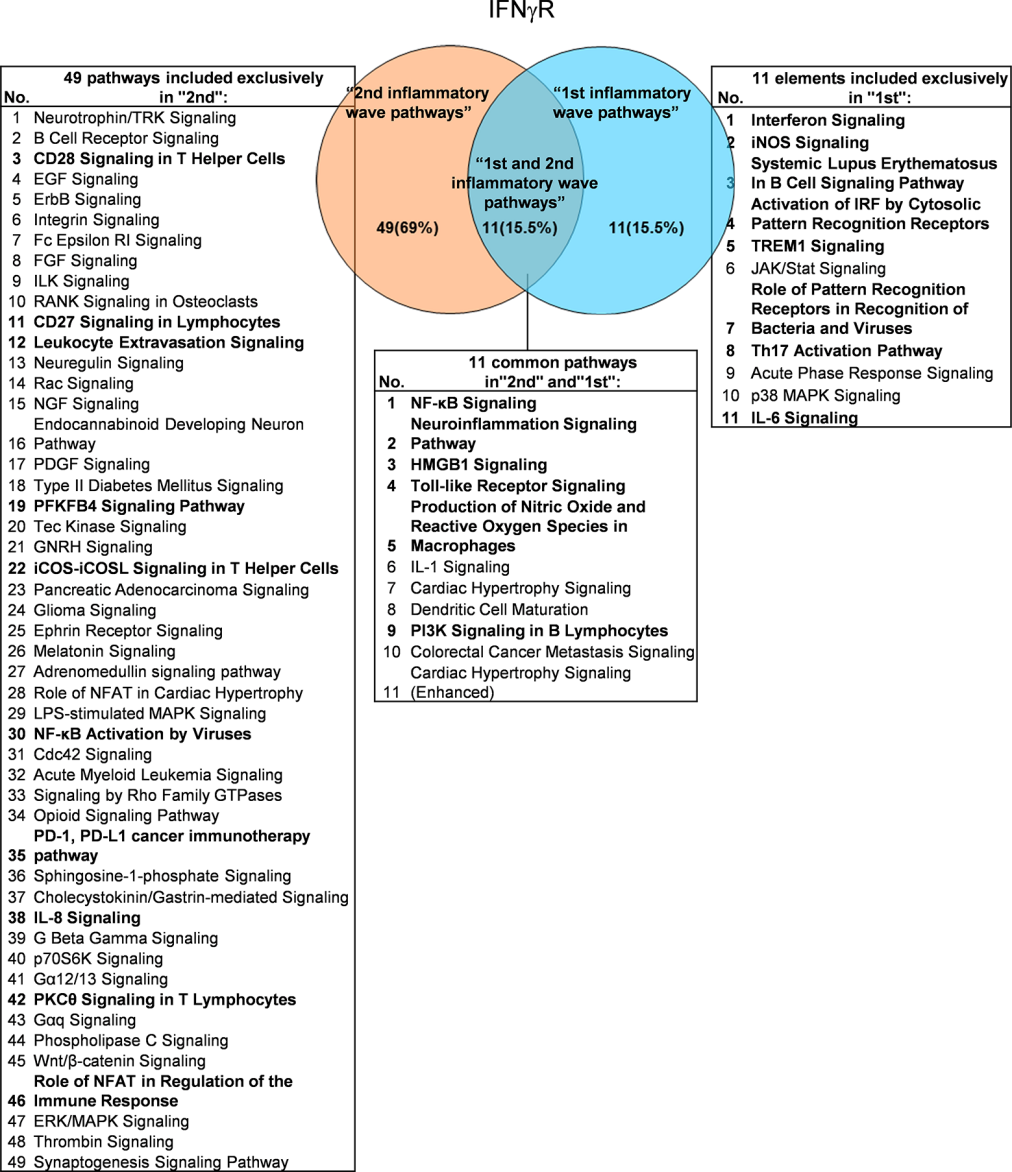
Venn diagram showing 11 overlapping significant pathways of up-regulated and down-regulated IGs in Ifngr KO microarray dataset (GSE39592). The data showed that in 11 common significant “2nd and 1st inflammatory wave pathways”, there were six cellular immune response related pathways. The data suggested that the up-regulated IGs have the same immune function to the down-regulated IGs by Ifngr KO. The suppression of PD-1, PD-L1 cancer immunotherapy pathway, and the activation of CD28 Signaling in T Helper Cells, CD27 Signaling in Lymphocytes, Leukocyte Extravasation Signaling, etc. ten pathways were unique cellular immune response pathways of “2nd inflammatory molecules”. Interferon Signaling, iNOS Signaling, Systemic Lupus Erythematosus etc. eight pathways were unique cellular immune response pathways of “1st inflammatory wave pathways”. The cellular immune response pathways were marked in bold.

**Figure 5:**
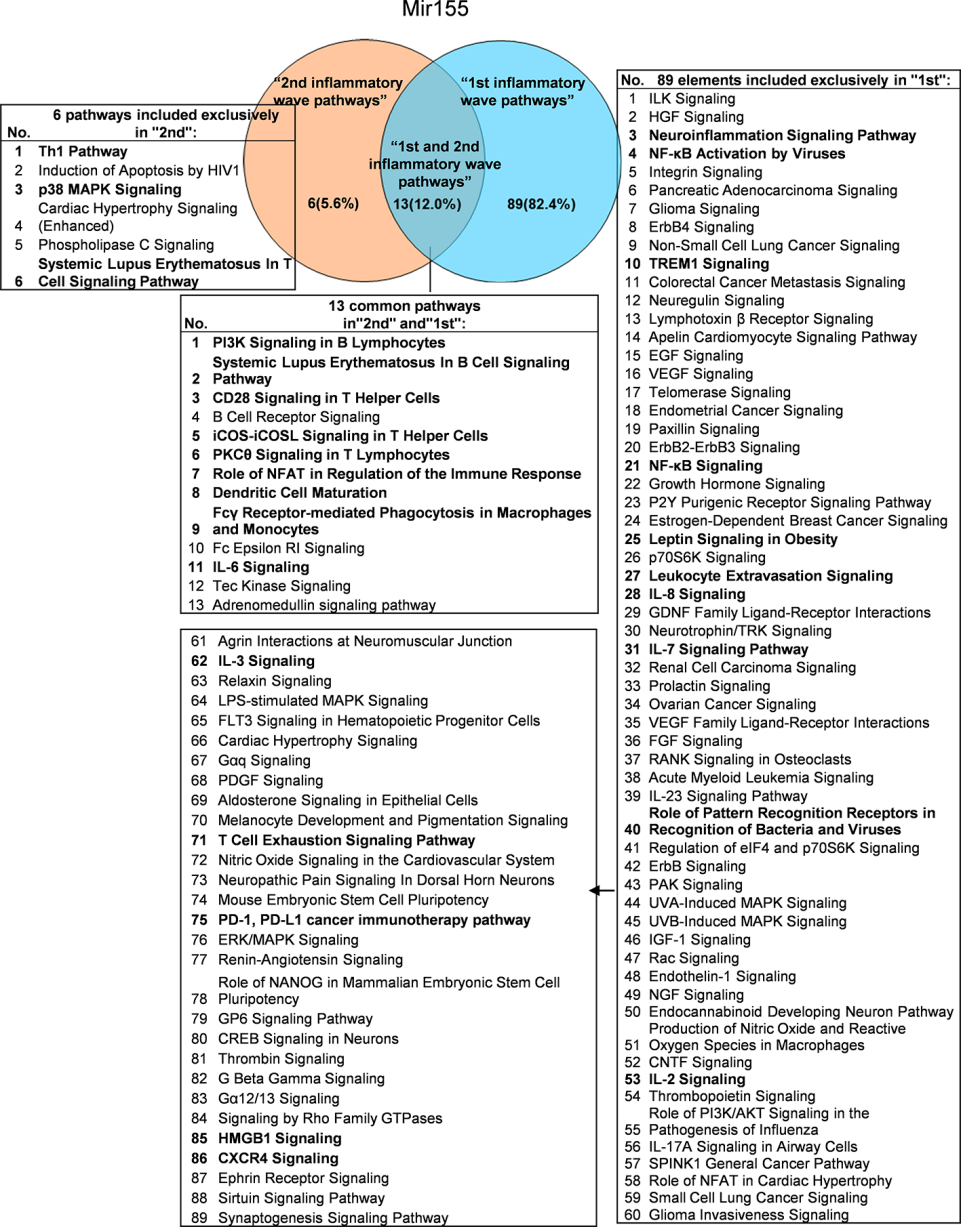
Venn diagram showing 13 overlapping significant pathways of up-regulated and down-regulated genes in mir155 KO microarray dataset (GSE66815). The data showed in 13 common significant “2nd and 1st inflammatory wave pathways”, there were nine cellular immune response related pathways. The data suggested that the up-regulated IGs have the same immune function to the down-regulated IGs by inflammatory miRNA155 KO. Six pathways were unique pathways of “2nd inflammatory molecules” and three pathways were cellular immune response related pathways. 89 pathways were unique pathways of “1st inflammatory molecules” and 15 pathways were cellular immune response related pathways. That is, the main significant pathways of miR155 are “1st inflammatory wave pathways”. And in “1st inflammatory wave pathways”, Leptin Signaling in Obesity was inhibited, which can explain the pathogenesis of MHO partly. The cellular immune response pathways were marked in bold.

It has been well documented that IFNγ secreted from Th1 cells and natural killer cells (NK) play proinflammatory roles (62), and miR155 have strong proinflammatory/proatherogenic roles (63). However, our recent papers reported that IFNγ and TNFα stimulated endothelial cells upregulate co-stimulation receptors B7-H2, CD40, SEMA4A (a member of the semaphorin family of soluble and transmembrane proteins) and CD112 and immune checkpoint receptors Galectin 9, herpesvirus entry mediator (HVEM), B7-DC, and B7-H1 (PD-L1) (64); proinflammatory adipokines leptin and resistin are significantly upregulated in the plasma and white adipose tissue in miR155 KO mice (31, 32). These findings suggest that IFNγ may inhibit endothelial cells activation and inflammation *via* anti-inflammatory reverse signaling mediated by upregulated immune checkpoint receptors (65), and miR155 may suppress the second wave of inflammation as formulated in our recently proposed new concept (32). We hypothesized that IFNγ-promoted and -suppressed innate immune programs have shared pathways; and miR155 promoted and suppressed innate immune programs have shared pathways. The rationale for us to focus on these two inflammatory regulators is that among the proinflammatory regulators examined (**Table 3A**), both these two have long lists of promoted and suppressed pathways. IFNγ KO mice have 37 specific down-regulated innatome pathways, seven specific up-regulated innatome pathways, and four common pathways shared by both promoted and suppressed programs including cardiac hypertrophy, integrin-linked kinase (ILK), role of NFAT in regulation of the immune response and colorectal cancer metastasis signaling (**Figure 3**). IFNγR1 KO mice have 11 specific downregulated innatome pathways, 49 specific upregulated innatome pathways and 11 common pathways shared by both IFNγR1-promoted and suppressed programs including NF-kB, neuroinflammation, high mobility group box 1 (HMGB1), Toll-like receptors, production of reactive nitric oxide and ROS in macrophages, IL-1, cardiac hypertrophy, dendritic cell maturation, PI3K signaling in B cells, colorectal cancer metastasis, and cardiac hypertrophy (enhanced) (**Figure 4**). Of note, in addition to IFNγR1/2, IFNγ has also been shown to signal through alternative pathways, including signal transducer and activator of transcription 4 (STAT4), extracellular signal-regulated protein kinases 1 and 2 (Erk1/2), proline-rich tyrosine kinase 2 (Pyk2), and CRK like proto-oncogene (CrkL), among others (66, 67), which may explain the discrepancies between IFNγ signaling and IFNγR1 signaling. In addition, miR155 KO have 89 specific downregulated pathways, six specific upregulated innatome pathways and 13 common pathways shared by both miR155-promoted and suppressed programs including PI3K signaling in B cells, systemic lupus erythematosus in B cell signaling, CD28 signaling in T helper cells, B cell receptor, inducible T-cell co-stimulator (iCOS)-iCOSL signaling, protein kinase C theta (PKCθ) signaling, role of nuclear factor of activated T cells (NF-AT), dendritic cell maturation, Fcγ receptor (one antibody receptor)-mediated phagocytosis, Fc Epsilon RI, IL-6, Tec kinase, and adrenomedullin signaling (**Figure 5**).

These results have demonstrated that: *first*, IFNγ promoted innate immune pathways (37 pathways) are more than that of IFNγ-suppressed pathways (7 pathways), suggesting that IFNγ plays roles in more upregulating IGs and less downregulating IGs; *second*, IFNγR1 promoted innate immune pathways (11 pathways) are less than that of IFNγR1-suppressed pathways (49 pathways), suggesting that IFNγR1 plays roles in less upregulating IGs and more downregulating IGs; *third*, miR155-promoted innate immune pathways (89 pathways) are more than that of miR155-suppressed pathways (6 pathways), suggesting that miR155 plays roles in more upregulating IGs and less downregulating IGs; *fourth*, IFNγ/IFNγR1 and miR155 both have their own promoted and suppressed pathways; and *fifth*, IFNγ/IFNγR1 has different shared pathways from that of miR155. These results have also suggested that proinflammatory molecules IFNγ and miR155 have negative-feedback mechanisms underlying downstream regulation.

### Deficiencies of PCs and Transcription Factors-Suppressed, -Promoted Programs Share the Signaling Pathways and Likelihood to Develop 11 Diseases, Including Cardiovascular Disease

**Figure 6:**
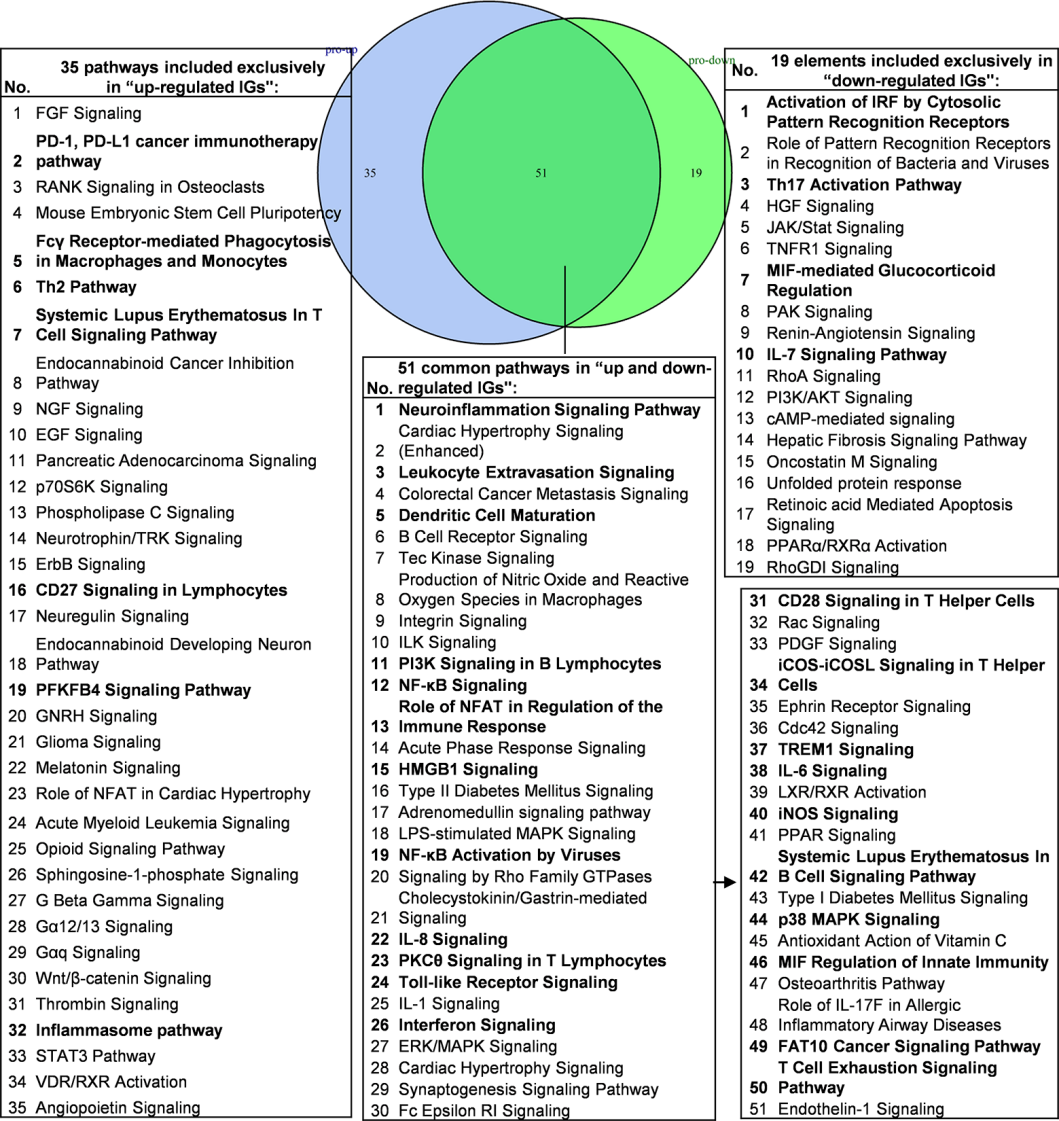
Venn diagram showing 51 overlapping significant pathways of up-regulated and down-regulated innatomic genes in proinflammatory cytokine KO microarray datasets. The significant pathways from down-regulated innate immune genes have the same expression pattern to proinflammatory cytokines. The data showed in 51 common significant pathways, there were about 22 cellular immune response related pathways (account for 43.14%). The data suggested that the up-regulated innate immune genes have the same immune function to the down-regulated innate immune genes by proinflammatory cytokines KO. Additionally, the suppression of PD-1, PD-L1 cancer immunotherapy pathway, Fcγ Receptor-mediated Phagocytosis in Macrophages and Monocytes, Th2 Pathway, Systemic Lupus Erythematosus In T Cell Signaling Pathway, CD27 Signaling in Lymphocytes, PFKFB4 Signaling Pathway and Inflammasome pathway were unique cellular immune response pathways of the up-regulated innate immunity genes after cytokines KO. Activation of IRF by Cytosolic Pattern Recognition Receptors, Th17 Activation Pathway, MIF-mediated Glucocorticoid Regulation, IL-7 Signaling Pathway were unique cellular immune response pathways of the down-regulated innate immunity genes after cytokines KO. The detailed IPA results were showed in **Table S7**, **S8**. The cellular immune response pathways were marked in bold.

We hypothesized that the common signaling pathways shared in PCs-promoted-, and suppressed programs play significant roles in the initiation and development of inflammation. To examine this hypothesis, we compiled all the significantly downregulated and upregulated pathways in the deficiencies of all the proinflammatory molecules examined (**Figure 6**). The results showed that proinflammatory molecules deficiencies downregulate 19 specific pathways such as activation of interferon regulatory factor (IRF) by cytosolic pattern recognition receptors, role of pattern recognition receptors in recognition of bacteria and viruses, Th17 activation, hepatocyte growth factor (HGF) signaling, Janus kinases (JAK)/STAT signaling, TNFR1 signaling, macrophage migration inhibitory factor (MIF)-mediated glucocorticoid regulation, p21-activated kinase (PAK) signaling, renin-angiotensin signaling, IL-7 signaling, RhoA signaling, phosphatidylinositol-3-kinase (PI3K)/protein kinase B (AKT) signaling and cyclic adenosine monophosphate (cAMP)-mediated signaling, suggesting that these signaling pathways are essential for promoting inflammation and anti-infection innate immune responses. In addition, we found that 35 pathways were associated with upregulated IGs when proinflammatory molecules are deficient, suggesting that the 35 pathways are often suppressed by proinflammatory molecules; these 35 pathways serve as the key players in the second wave of inflammation when these PCs and molecules are deficient. Moreover, we identified 51 common pathways in IGs that are shared by proinflammatory molecules-promoted and suppressed programs, suggesting that these 51 common pathways allow the launching of effective inflammation, and innate and adaptive immune responses in the presence and absence of individual proinflammatory molecules. These common pathways are novel targets for future therapeutics to make proinflammatory cytokine blockade therapy more effective.

**Figure 7:**
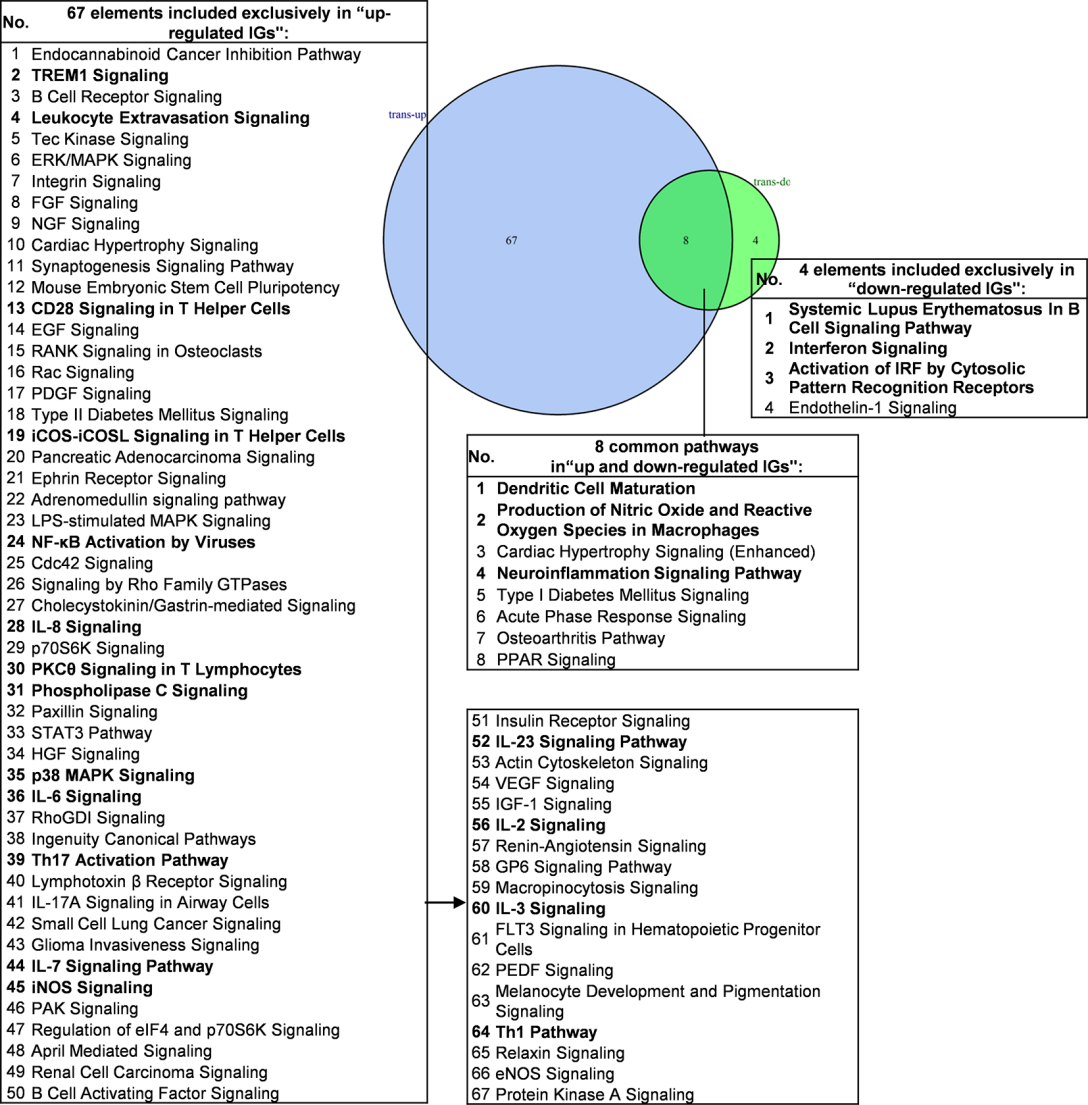
Venn diagram showing eight overlapping significant pathways of up-regulated and down-regulated innatomic genes in pro-inflammatory related transcription factor KO microarray datasets. The significant pathways from down-regulated innate immune genes have the same expression pattern to proinflammatory related transcription factors. The data showed that in 8 common significant pathways, there were 3 cellular immune response related pathways. The data suggested that the up-regulated innate immune genes have the same immune function to the down-regulated innate immune genes by proinflammatory related transcription factors KO. There are 67 significant pathways in up-regulated innate immune genes including TREM1 Signaling, Leukocyte Extravasation Signaling, CD28 Signaling in T Helper Cells etc. cellular immune response pathways. The cellular immune response pathways were marked in bold. The detailed IPA results were showed in **Tables S7**–**S9**. The cellular immune response pathways were marked in bold.

We then hypothesized that the common signaling pathways shared in proinflammatory transcription factors (TFs) KO, including STAT1, STAT3, NF-KB Rela and IKK2-promoted-, and suppressed programs play significant roles on initiation and development of inflammation. To examine this hypothesis, we compiled all the significantly downregulated and upregulated pathways in the deficiencies of all the proinflammatory TFs (**Figure 7**). The results showed that proinflammatory TFs deficiencies-downregulate four pathways (systemic lupus erythematosus in B cell signaling, interferon signaling, activation of IRF by cytosolic pattern recognition receptors, and endothelin-1 signaling) suggesting that these four pathways are promoted by the four TFs. In addition, we found 67 pathways in the IGs upregulated in the four TF deficiencies, suggesting that these four TFs suppress a long list of innate immune pathways. Moreover, we found that eight pathways are shared in the IGs promoted and suppressed when the four TFs are deficient (**Table S9** lists pathways of up-regulated IGs and down-regulated IGs in each microarray). Taken together, PCs-promoted and -suppressed innate immune programs have 51 shared pathways; and proinflammatory transcription factors suppress 67 innate immune pathways, which may become novel targets for the future therapeutics and also suggest strategical problems in targeting proinflammatory transcription factors for therapies.

**Figure 8:**
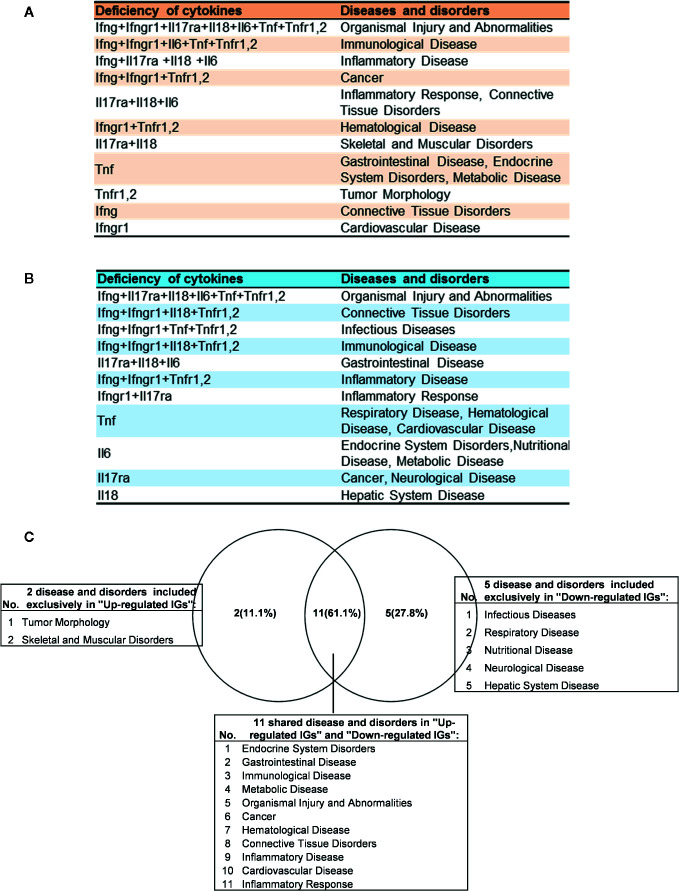
The up- and down-regulated innatomic genes (IGs) share inflammatory diseases in deficiencies of proinflammatory cytokines (PCs). **(A)** The upregulated IGs in PCs KO microarrays share 8 different diseases and disorders including inflammatory diseases. **(B)** The down-regulated IGs in PCs KO microarrays share 7 different diseases and disorders including infectious diseases (increased) and inflammatory diseases. **(C)** Venn diagram showed the upregulated IGs and the downregulated IGs share 11 diseases or disorders, two diseases or disorders included exclusively in “Up-regulated IGs” and five disease or disorders included exclusively in “Down-regulated IGs”. In them, cardiovascular disease is shared in up- and down-regulated IGs.

As shown in **Table 4** and **Figure 8A**, the results demonstrated a range from multiple cytokine deficiencies to single cytokine deficiency that: *1)* deficiencies of all seven cytokines including IFNγ, IFNγR1, IL17RA, IL18, IL6, TNFα, and TNFR1,2 upregulated organismal injury and abnormalities; *2)* deficiencies of five cytokines IFNγ, IFNγR1, IL6, TNFα, and TNFR1,2 upregulated immunological diseases; *3)* deficiencies of four cytokines IFNγ, IL17ra, IL18, and IL6 upregulated inflammatory diseases; deficiencies of three cytokines IFNγ, IFNγR1, TNFR1,2 upregulated cancer; *4)* deficiencies another three cytokines IL17RA, IL18 and IL6 upregulated inflammatory response, connective tissue disorders; *5)* deficiencies of two cytokines IFNγR1 and TNFR1,2 upregulated hematological disease; *6)* deficiencies of another two cytokines IL17ra and IL18 upregulated skeletal and muscular disorders; *7)* deficiency of cytokine TNF upregulated gastrointestinal disease, endocrine system disorders and metabolic disease; *8)* deficiency of cytokine TNFR1,2 upregulated tumor morphology; *9)* deficiency of cytokine IFNγ upregulated connective tissue disorders; and *10)* deficiency of cytokine receptor IFNγR1 increase cardiovascular disease. Similarly, we performed IPA for top disease and disorder associations of downregulated IGs in cytokine gene KO microarrays as shown in **Table 4** and **Figure 8B**. The results are significant since some cytokine blockage therapies have not used for treatment of those top diseases yet. The results demonstrated that each cytokine KO decreased the likelihood to develop certain diseases, which provide novel valuable guidance for cytokine blockage therapies based on the cytokine modulating effects on the expression of IGs.

**Table 4:** The top 5 disease and disorders of the up-regulated and down-regulated IGs in cytokines KO microarrays.

PCs (upN/downN)	Up-regulated IGs			Down-regulated IGs		
	Top Diseases and disorders			Top Diseases and disorders		
	Name	p-value range	# Molecules	Name	p-value range	# Molecules
**Tnf KO**						
(52/24)	Endocrine System Disorders	5.47E-04 - 1.07E-15	25	Infectious Diseases	3.34E-02 - 2.12E-05	4
	Gastrointestinal Disease	5.77E-04 - 1.07E-15	29	Organismal Injury and Abnormalities	3.60E-02 - 2.12E-05	22
	Immunological Disease	7.06E-04 - 1.07E-15	36	Respiratory Disease	3.34E-02 - 2.12E-05	4
	Metabolic Disease	3.18E-04 - 1.07E-15	21	Cardiovascular Disease	3.04E-02 - 7.84E-05	7
	Organismal Injury and Abnormalities	7.69E-04 - 1.07E-15	44	Hematological Disease	2.54E-02 - 7.84E-05	5
**Tnfr1,2 KO**	Immunological Disease	4.24E-03 - 1.49E-07	18	Immunological Disease	1.04E-08 - 1.62E-39	113
(32/171)	Cancer	4.24E-03 - 5.41E-07	28	Infectious Diseases	1.07E-08 - 2.19E-33	87
	Hematological Disease	4.24E-03 - 5.41E-07	19	Connective Tissue Disorders	8.87E-09 - 1.04E-29	76
	Organismal Injury and Abnormalities	4.24E-03 - 5.41E-07	28	Inflammatory Disease	5.62E-09 - 1.04E-29	92
	Tumor Morphology	4.24E-03 - 9.23E-07	4	Organismal Injury and Abnormalities	1.18E-08 - 1.04E-29	152
**Ifng KO**	Cancer	5.81E-04 - 8.88E-09	79	Immunological Disease	3.22E-08 - 2.68E-38	97
(81/138)	Organismal Injury and Abnormalities	5.81E-04 - 8.88E-09	79	Infectious Diseases	2.26E-08 - 1.47E-34	82
	Immunological Disease	5.81E-04 - 1.00E-08	43	Connective Tissue Disorders	4.62E-08 - 5.68E-33	69
	Connective Tissue Disorders	5.81E-04 - 1.47E-08	26	Inflammatory Disease	3.11E-08 - 5.68E-33	78
	Inflammatory Disease	4.24E-04 - 1.47E-08	32	Organismal Injury and Abnormalities	4.62E-08 - 5.68E-33	131
**Ifngr1 KO**	Organismal Injury and Abnormalities	1.49E-04 - 1.51E-14	105	Infectious Diseases	2.01E-05 - 1.37E-21	46
(111/82)	Hematological Disease	1.18E-04 - 9.22E-11	50	Immunological Disease	1.35E-05 - 9.81E-21	51
	Immunological Disease	1.31E-04 - 9.22E-11	44	Inflammatory Response	1.92E-05 - 2.13E-19	54
	Cardiovascular Disease	1.46E-04 - 1.04E-10	29	Connective Tissue Disorders	1.48E-05 - 2.82E-19	40
	Cancer	1.49E-04 - 1.19E-10	103	Inflammatory Disease	1.65E-05 - 2.82E-19	50
**Il6 KO**	Inflammatory Response	1.39E-03 - 3.58E-17	34	Endocrine System Disorders	6.76E-03 - 1.87E-06	5
(44/13)	Immunological Disease	1.23E-03 - 2.76E-12	35	Gastrointestinal Disease	6.38E-03 - 1.87E-06	13
	Organismal Injury and Abnormalities	1.40E-03 - 1.16E-10	40	Metabolic Disease	7.38E-03 - 1.87E-06	7
	Connective Tissue Disorders	1.23E-03 - 2.07E-10	22	Nutritional Disease	5.22E-03 - 1.87E-06	4
	Inflammatory Disease	1.23E-03 - 2.07E-10	27	Organismal Injury and Abnormalities	7.54E-03 - 1.87E-06	13
**Il17ra KO**	Inflammatory Response	7.74E-12 - 1.20E-39	100	Inflammatory Response	4.47E-03 - 3.68E-06	21
(141/50)	Connective Tissue Disorders	5.90E-12 - 1.96E-37	72	Neurological Disease	4.47E-03 - 1.07E-05	21
	Inflammatory Disease	5.33E-12 - 1.96E-37	83	Organismal Injury and Abnormalities	4.47E-03 - 1.07E-05	49
	Organismal Injury and Abnormalities	1.52E-11 - 1.96E-37	115	Cancer	4.37E-03 - 1.21E-05	48
	Skeletal and Muscular Disorders	2.96E-12 - 1.96E-37	78	Gastrointestinal Disease	4.47E-03 - 1.21E-05	46
**Il18 KO**	Connective Tissue Disorders	6.58E-04 - 5.71E-10	22	Gastrointestinal Disease	1.12E-02 - 1.32E-06	6
**(adipose)**	Inflammatory Disease	1.80E-04 - 5.71E-10	22	Hepatic System Disease	3.22E-03 - 1.32E-06	4
(48/12)	Inflammatory Response	6.55E-04 - 5.71E-10	28	Organismal Injury and Abnormalities	1.12E-02 - 1.32E-06	11
	Organismal Injury and Abnormalities	7.20E-04 - 5.71E-10	46	Connective Tissue Disorders	9.09E-03 - 7.38E-06	7
	Skeletal and Muscular Disorders	6.45E-04 - 5.71E-10	24	Immunological Disease	1.02E-02 - 7.38E-06	6

upN/down: number of up-regulated IGs/number of down-regulated IGs.

Of note, as shown in **Figure 8C**, comparing cytokine deficiencies-downregulated IGs related diseases to that of cytokine deficiencies-upregulated IGs related diseases, the results showed that *first*, the cytokine deficiencies-downregulated IGs related disease group has five diseases including infectious disease (increased, **Figure S1**), respiratory disease, nutritional disease, neurological disease, and hepatic system disease; *second*, cytokine deficiencies-upregulated IGs related disease group has two diseases such as tumor morphology and skeletal and muscular disorders; and *third*, the two groups share 11 diseases, suggesting that current therapeutic strategies have significant problems; and there are urgent needs to re-shape the strategies in designing cytokine blockage therapies. Taken together, our results have demonstrated that *first*, the deficiencies of PCs not only upregulate IGs and innate immune signaling pathways but also increase the likelihood to develop certain immune, inflammatory diseases and cancers; and *second*, the deficiencies of PCs not only downregulate IGs and innate immune signaling pathways but also decrease the likelihood to develop certain immune, inflammatory diseases and cancers and provide novel valuable guidance for cytokine blockage therapies.

### Deficiency of TNFα in Mice and Patients Receiving Anti-TNF Therapy Shared Upregulated IGs and Inflammatory Pathways

**Figure 9:**
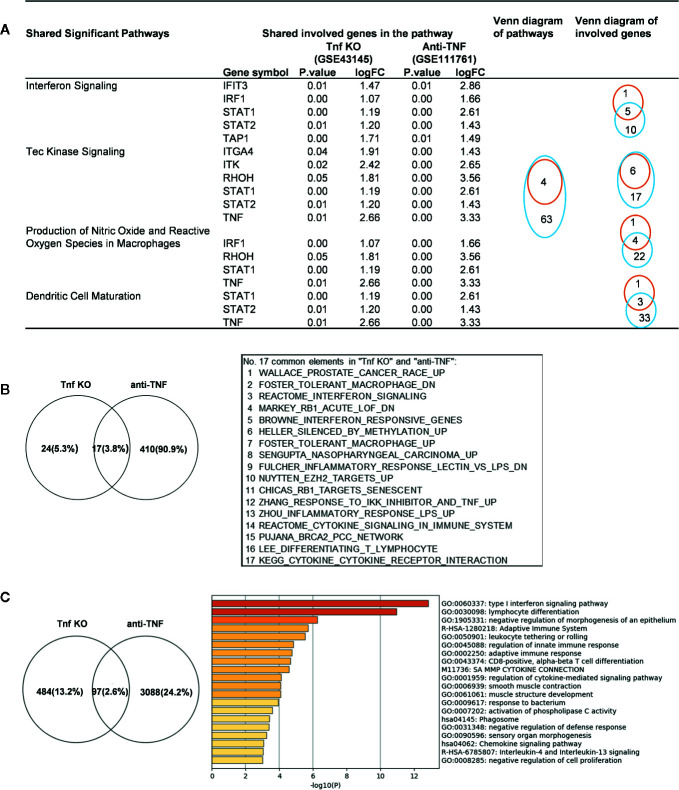
Up-regulated innatomic genes (IGs) in Tnf KO (GSE43145, Tnf KO Gan mice versus (vs.) Gan mice) and anti-TNF therapy (GSE111761, non-responder vs. responder) microarrays shared pathways. **(A)** IPA results of the pathways and the involved genes from innatome were compared. Some genes were involved more than two pathways. Orange line represents Tnf KO and blue line represents Anti-TNF therapy. **(B)** All the significant differential expressed genes (*p*<0.05, ∣logFC∣>1) of these two microarrays were enrichment analyzed by using GSEA. The result showed a total of 17 pathways were shared by Tnf KO and anti-TNF therapy microarrays. The result showed that except for interferon signaling, several cancer related, inflammatory response and cytokine signalings, were activated in these two microarrays. **(C)** Venn diagram showed 97 significantly differentially expressed genes were shared by Tnf KO and anti-TNF therapy microarrays. The 97 genes were carried out enrichment analysis by using metascape. The result showed the type I interferon signaling pathway, lymphocyte differentiation, Adaptive Immune System, regulation of innate immune response, response to bacterium etc. were the significantly enriched GO or pathways. **Table S10** is IPA results of up- and down-regulated innatomic genes in anti-TNF therapy microarray.

We made an interesting finding that, the IGs were more upregulated than downregulated when poor responder or non-responder compared with the group responders or good responders in several microarray datasets of patients receiving monoclonal antibody (Mab) therapy (**Table 2B**). Then, we hypothesized that in drug none-responders, “suppressed cytokines or innate immune regulator molecules” were upregulated. To examine this hypothesis, Tnf KO (GSE43145, Tnf KO Gan mice vs. Gan mice) and anti-TNF therapy (GSE111761, non-responders vs. responders) microarrays were compared. The IPA results of the pathways and the involved IGs showed that four upregulated pathways in Tnf KO microarray also were all shared with that in patients receiving anti-TNF therapy; and several upregulated IGs involved in the pathways were shared (**Figure 9A**). IPA results of up-regulated innatomic genes in anti-TNF therapy microarray were listed in **Table S10**. All the significant differential expressed genes (*p*<0.05, ∣logFC∣>1) of these two microarrays were further analyzed by using GSEA for pathway enrichments (68, 69). The result showed that a total of 17 pathways were shared by Tnf KO and anti-TNF therapy microarrays including inflammatory response and cytokine signalings, which were activated in these two microarrays (**Figure 9B**). The Venn diagram showed that 97 significant differentially expressed IGs were shared by Tnf KO and anti-TNF therapy microarrays. The 97 genes were analyzed for additional enrichment analysis by using the Metascape database. The result showed that the five pathways such as type I interferon signaling pathway, lymphocyte differentiation, Adaptive Immune System, regulation of innate immune response, response to bacterium etc, were the significantly enriched GO or pathways (**Figure 9C**). Taken together, based on not only from the IPA results of significant differentially expressed IGs, but also the integrated analysis results from the GSEA and Metascape of all shared significant differentially expressed genes, our analyses have demonstrated that the new inflammatory responses will be activated when TNF is suppressed, thus supporting our novel hypothesis that the “2nd inflammatory wave” contributes to non-responder patients receiving anti-TNF Mab therapy partially. In fact, our findings were well correlated with the report about GSE111761 that in patients with Crohn’s disease receiving anti-TNF therapy, there were significant upregulations of mucosal Il-23p19, Il23R and Il17A in non-responders, but not in responders.

### Up-Regulated ROS Regulators Such as MPO Caused by Suppression of Major Proinflammatory Molecules Can Drivethe Upregulation of “Suppressed Innatomic Genes”

**Figure 10:**
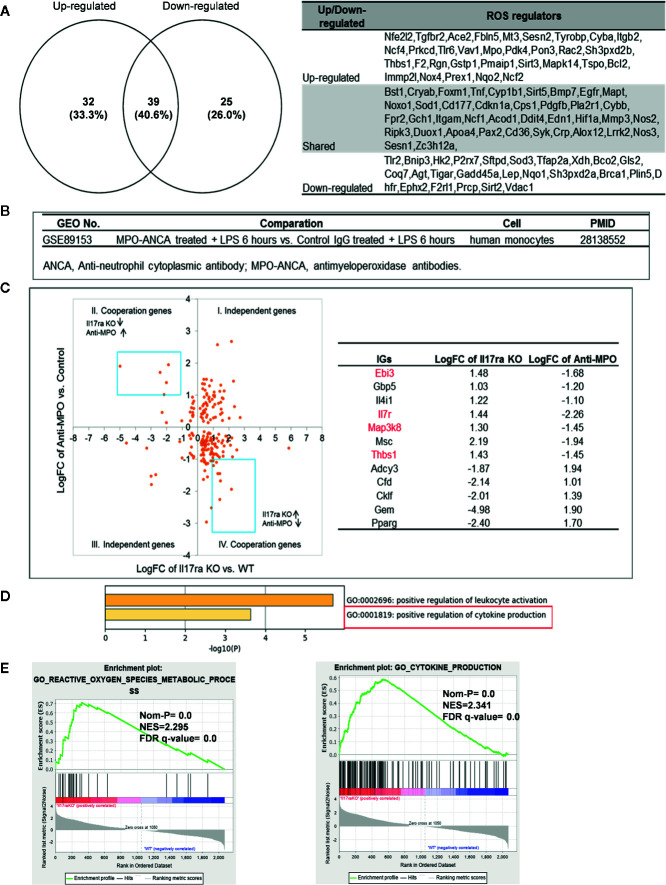
Mechanism: up-regulated reactive oxygen species (ROS) regulators can drive the upregulation of “suppressed innatomic genes”. **(A)** 32 ROS regulators were up-regulated, 25 ROS regulators were down-regulated and 39 ROS regulators were common in up-regulated and down-regulated. (*p*<0.05, ∣log2FC∣>1). **Table S11** listed the detailed expression changes of ROS regulators. **(B)** Anti-myeloperoxidase (MPO) antibodies microarray GSE89153 was searched in GEO database to analyze the cooperation between ROS regulator MPO and proinflammatory cytokine Il17ra. **(C)** Cooperation analysis shows 12 genes were highly cooperatively regulated by MPO and Il17ra (Blue line box, *p*<0.05, ∣log2FC∣>1). **(D)** GO enrichment analyzed from Metascape software showed four in seven down-regulated genes (red in **(b)** were enriched in GO:0001819: positive regulation of cytokine production. **(E)** GSEA results of all the significant differential expression genes (*p*<0.05, ∣log2FC∣>1) in Il17ra KO showed the REACTIVE_OXYGEN_SPECIES_METABOLIC_PROCESS and POSITIVE_REGULATION_OF_CYTOKINE_PRODUCTION genes significantly enriched in the Il17ra KO groups. NES, Normalized Enrichment Score; FDR, false discovery rate.

Mitochondrial ROS (mtROS) are signaling molecules, which drive inflammatory cytokine production (70) and T cell activation (70, 71). In addition, CVDs, cancers, and autoimmune diseases all share a common feature of increased mtROS levels (72). Our recent study shed light on this important question and found that, during endothelial cell activation, mtROS could be upregulated in a proton leak-coupled, but ATP synthesis-uncoupled manner (72–75). As a result, endothelial cells could upregulate mtROS production for physiological endothelial cell activation without compromising mitochondrial membrane potential and ATP generation, and consequently without causing mitochondrial damage and endothelial cell death. Thus, a novel pathophysiological role of proton leak in driving mtROS production was uncovered for low-grade endothelial cell activation, patrolling immunosurveillance cell trans-endothelial migration and low-grade chronic inflammation without compromising cellular survival (72–76). One of the most evident features of the inflammatory response is the generation of a pro-oxidative environment due to the production of high fluxes of pro-oxidant species (77). We hypothesized that deficiencies of PCs and regulators upregulate some oxidative stress regulators. To test this hypothesis, we examined 165 ROS regulators collected in the Gene Set Enrichment Analysis (GSEA) database (https://www.gsea-msigdb.org/gsea/index.jsp). As shown in **Table 5**, 96 ROS regulators were modulated by deficiencies of PCs, transcription factors and miRs. **Table S11** listed the detail expression changes of ROS regulator. Deficiencies of IL6, STAT1, NF-kB-Rela, miR155 resulted in no downregulation of ROS regulators. Venn Diagram analysis (**Figure 10A**) showed that: *1)* deficiencies of proinflammatory regulators caused downregulation of 25 ROS regulators; *2)* deficiencies of proinflammatory regulators caused upregulation of 32 ROS regulators; and *3)* 39 ROS regulators were shared by upregulated and downregulated in deficiencies of proinflammatory regulators, suggesting that these 39 ROS regulators are required for the functions of modulated IGs in regardless of expressional levels of 9 proinflammatory regulators. Of note, the rest of the 69 ROS regulators were not significantly modulated in the deficiencies of major proinflammatory regulators.

**Table 5:** 165 ROS regulators were analyzed in inflammatory molecules KO microarrays.

No.	PCs and regulators	GEO NO.	Method	ROS regulators					
				Up-regulated					Down-regulated
				N	%	Genes	N	%	Genes
	**Proinflammatory cytokines**								
1	TNF	GSE43145	Tnfa KO	3	2.42	Bst1,Cryab,Foxm1	4	2.42	Ncf1,Noxo1,Sod1,Tlr2
2		GSE33253	Tnfr1,2 KO	4	2.42	Cyp1b1,Nfe2l2,Sirt5,Tgfbr2	13	7.88	Acod1,Cd36,Cps1,Crp,Cybb,Ddit4, Edn1,Fpr2,Mmp3,Ncf1,Pdgfb,Sesn1,Tnf
3	IFNG	GSE9892	Ifng KO	7	4.24	Ace2,Bmp7,Egfr,Fbln5,Mapt, Noxo1,Sod1	14	8.48	Acod1,Apoa4,Bnip3,Bst1,Cybb, Fpr2,Hk2,Nos2,P2rx7,Sftpd,Sod3, Tfap2a,Xdh,Zc3h12a
4		GSE39592	Ifngr1 KO	10	6.06	Cd177,Cdkn1a,Cps1,Cyp1b1, Mapt,Mt3,Pdgfb,Pla2r1,Sesn2, Sod1	11	6.67	Acod1,Bco2,Bmp7,Bst1,Cryab, Foxm1,Fpr2,Gls2,Ncf1,P2rx7,Sirt5
5	IL1B	GSE15750	Traf6 KO	0	0.00	N/A	2	1.21	Coq7,Syk
6		GSE73875	Irak1 KO	3	1.82	Cybb,Fpr2,Tyrobp	0	0.00	N/A
7	IL6	GSE63761	Il6 KO	11	6.67	Cyba,Gch1,Itgam,Itgb2,Ncf1, Ncf4,Nfe2l2,Prkcd,Tlr6,Tyrobp, Vav1	3	1.82	Agt,Ddit4,Nos3
8	IL17	GSE88800	Il17ra KO	23	13.94	Acod1,Cd177,Cdkn1a,Ddit4, Edn1,Fpr2,Gch1,Hif1a,Itgam, Mmp3,Mpo,Mt3,Ncf1,Ncf4, Nos2,Pdk4,Pon3,Rac2,Ripk3, Sh3pxd2b,Thbs1,Tlr6,Tnf	3	1.82	Bco2,Mapt,Tigar
9	IL18	GSE64308	Il18 KO	6	3.64	Cdkn1a,Duox1,Edn1,F2, Rgn,Thbs1	1	0.61	Alox12
10		GSE64309	Il18 KO	7	4.24	Apoa4,Cdkn1a,Ddit4,Gstp1, Mpo,Pax2,Pdk4	8	4.85	Bco2,Bnip3,Cd36,Duox1,Gadd45a, Itgam,Lep,Nqo1
11		GSE64310	Il18 KO	1	0.61	Apoa4	2	1.21	Bco2,Sh3pxd2a
	**ProInflammatory related transcription factor**								
12	STAT	GSE40666	Stat1 KO	6	3.64	Cd36,Cdkn1a,Mt3,Pmaip1, Sirt3,Syk	0	0.00	N/A
13		GSE6846	Stat3 KO	8	4.85	Cybb,Hif1a,Itgam,Mapk14, Ncf1,Pla2r1,Syk,Tyrobp	3	1.82	Brca1,Plin5,Sod1
14	NFKB	GSE45755	Rela KO	6	3.64	Cybb,Mpo,Ncf1,Ncf4,Tspo, Tyrobp	0	0.00	N/A
15		GSE30049	Ikk2 KO	9	5.45	Bcl2,Bmp7,Cryab,Cyp1b1, Immp2l,Nox4,Pdk4,Prex1, Thbs1	12	7.27	Brca1,Cybb,Dhfr,Edn1,Ephx2,F2rl1, Gch1,Nos2,Pdgfb,Prcp,Ripk3,Sod3
	**Inflammatory-related miRNAs**								
16	MIR155	GSE45122	mir155 KO	3	1.82	Bmp7,Crp,Nqo2	3	1.82	Cyp1b1,Lrrk2,Pax2
17		GSE66815	mir155 KO	13	7.88	Alox12,Bst1,Cyp1b1,Lrrk2, Ncf2,Nos3,Pmaip1,Prex1,Rac2,Sesn1,Syk,Vav1,Zc3h12a	10	6.06	Agt,Cd177,Cdkn1a,Egfr,Foxm1, Hif1a,Noxo1,Nqo1,Sirt2,Vdac1
18	MIR221	GSE19777	MIR221 KD	1	0.61	CPS1	4	2.42	ACOD1,CYBB,PLA2R1,SFTPD

**Table S11** listed the detail expression changes of ROS regulator. (p < 0.05, ∣log2FC∣>1).

Our detailed results showed that deficiencies of IL17RA, IL18 and NF-kB Rela result in upregulated heme peroxidase myeloperoxidase (MPO) expression (78). MPO and MPO-derived oxidants have been shown to contribute to the formation of foam cells, endothelial dysfunction and apoptosis, the activation of latent matrix metalloproteinases, and the expression of tissue factor that can promote the development of vulnerable plaque. Then, we hypothesized that IGs upregulated in deficiency of IL17RA are also upregulated by MPO. To examine this hypothesis, we found an anti-MPO antibodies microarray GSE89153 (**Figure 10B**) from the NIH-GEO database to analyze the cooperation between ROS generator MPO (**Figure 10C**) and proinflammatory cytokine receptor IL17RA. The cooperation analysis, as we reported (55), showed that among 197 common significantly differentially expressed IGs in IL17RA KO and Anti-MPO microarrays, 121 IGs (61.42%) were cooperatively regulated (*p*<0.05). 12 genes were highly cooperatively regulated (Blue line box, *p*<0.05, ∣log2FC∣>1). The GO enrichment (http://geneontology.org/) was analyzed by using Metascape software and GSEA (68, 69). The result showed that four in seven down-regulated genes (marked in red in **Figure 10C**) were enriched in GO:0001819: positive regulation of cytokine production (**Figure 10D**). The GSEA results of all the significant differential expression genes (*p*<0.05, ∣log2FC∣>1) in IL17RA KO showed the REACTIVE_OXYGEN_SPECIES_METABOLIC_PROCESS and POSITIVE_REGULATION_OF_CYTOKINE_PRODUCTION genes significantly enriched in the IL17RA KO groups with notable normalized enrichment score (NES; https://en.wikipedia.org/wiki/Gene_set_enrichment_analysis) and false discovery rate (FDR; https://en.wikipedia.org/wiki/False_discovery_rate) (**Figure 10E**). Our results have demonstrated a novel mechanism that deficiencies of IL-17RA and presumably IL-18 promote MPO upregulation, which drives a ROS-dependent inflammation.

## Discussions

These progresses lead to the development of many cytokine blockage-based therapies for inflammatory diseases and CVDs. The CANTOS trial with the Mab Canakinumab to block proinflammatory cytokine IL-1β was a recent success in treating coronary artery disease (30). However, recent reports from our and others’ teams suggest that inhibition of one proinflammatory regulators such as cytokines or microRNAs leads to new waves of inflammation. To approach these types of inflammation paradoxes, we performed an extensive -omics data mining analyses with the method that we pioneered in 2004 (56, 57, 79) and made a set of significant findings: *1)* PCs suppress IGs; and upregulated IGs in the deficiencies of IFNγ, IFNγR1, IL-17A, STAT3 and miR155 are more than that after deficiencies of TNFα, IL-1β, IL-6, IL-18, STAT1, NF-kB, and miR221; *2)* IFNγ/IFNγR1 and IL-17RA inhibit 10, 59 and 39 pathways, respectively; in contrast TNFα, IL-6 and IL-18 inhibits four to five pathways; *3)* The IFNγ-promoted and -suppressed programs have 4 shared pathways, IFNγR1-promoted and -suppressed programs have 11 shared pathways; and miR155-promoted and -suppressed programs have 13 shared pathways, suggesting negative-feedback mechanisms in their regulatory pathways for IGs; *4)* Deficiencies of PCs and transcription factors-suppressed, -promoted programs share the signaling pathways and likelihood to develop 11 diseases including cardiovascular disease; *5)* There are shared IGs and pathways between deficiency of TNFα in mice and anti-TNF therapy in clinical patients; *6)* Mechanistically, up-regulated ROS regulators such as MPO caused by suppression of major proinflammatory molecules can drive the upregulation of “suppressed IGs”.

The original microarray experiments used different cells, which prevented us from comparing the effects of proinflammatory regulators in regulating the expressions of IGs in the same cell types. Although our database mining approach was not ideal, however, as the first step to fill in the important knowledge gap this approach was justified. Actually, this was a common practice that we (50) and others (51) often used in studying gene expression in non-ideal, heterogenous peripheral blood mononuclear cell populations (PBMCs) in pathophysiological conditions versus healthy conditions, which are actually composed of many cell types, such as B cells (~15 %), T cells (~70 %), monocytes (~5 %), and natural killer (NK) cells (~10 %) among others (80).

Indeed, in addition to PC regulation of IGs transcription examined in our study, PCs could also regulate innate immune regulators in several other modes: 1) mRNA stability (81); 2) riboclustering (82); 3) alternative splicing (83); 4) microRNA regulation (84); 5) long non-coding RNA regulation (85); 6) circular RNAs regulation (86); 7) protein translation (87); 8) protein neddylation (88); 9) ubiquitination-proteasome regulation (89); 10) epigenetic regulation (90); and 11) immune metabolism and innate immune memory (91, 92). The proof of principles demonstrated in examination of inflammatory paradoxes performed in the current manuscript are far-reaching and can be extended to other fields of study such as cancers, infectious diseases such as COVID-19 and various pathologies.

**Figure 11:**
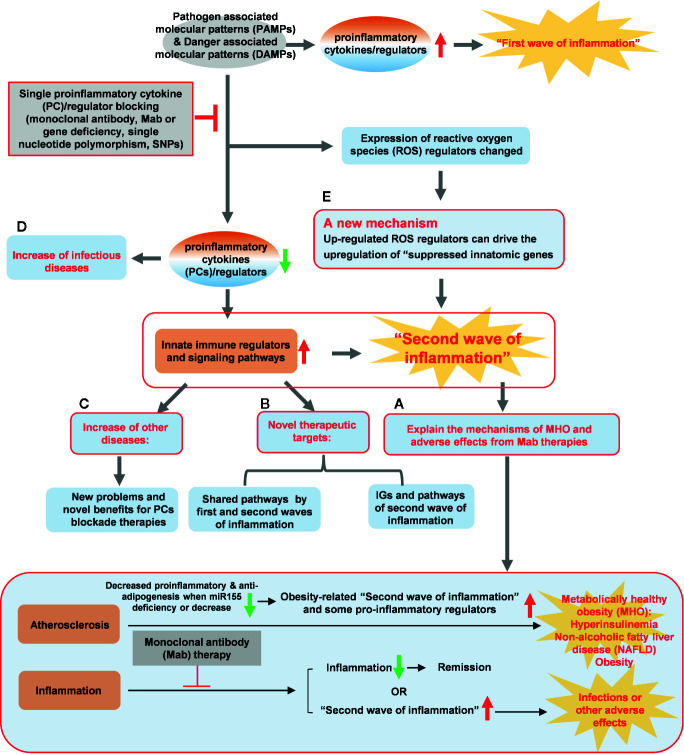
A new working model is proposed: Blocking proinflammatory regulators induces the proinflammatory regulators-suppressed “second waves of inflammation”. Single cytokine blockade therapies result in significant upregulation of innate immune regulators and signaling pathways, presumably “second wave of inflammation” as we proposed. **(A)** The second wave of inflammation may be the underlying mechanisms for metabolically healthy obesity (MHO), adverse effects observed in patients receiving monoclonal antibody (Mab) therapies in blocking proinflammatory cytokines. **(B)** The two groups of new innatome genes (IGs) and their pathways have been identified as novel therapeutic targets. **(C)** Deficiencies of proinflammatory cytokines and regulators upregulate innate immunomic genes reveal new problems and novel benefits for proinflammatory cytokine (PCs) blockade therapies. **(D)** Down-regulated IGs can increase infectious diseases. **(E)** PCs blockade can regulate the expression of ROS regulators and up-regulated ROS regulators can upregulate expression of IGs. A new mechanism, ROS regulators, has been identified for modulating the expressions of IGs carrying out the second wave of inflammation.

To summarize our findings presented here, we propose a novel working model (**Figure 11**) to integrate these results as follows: *first*, since proinflammatory cytokines and regulators are interconnected through evolution, single cytokine blockade therapies result in significant upregulation of a long list of IGs and signaling pathways, presumably the “second wave of inflammation” as we proposed (32). The second wave of inflammation suppressed by PCs and regulators has never been identified in this comprehensive manner. The second wave of inflammation may be the underlying mechanisms for MHO, adverse effects observed in patients receiving Mab therapies blocking PCs. Novel therapeutic strategies need to be developed based on our findings to combine several related immune therapies as demonstrated by the synergy between immune checkpoint receptor CD279 blocking therapy and anti-TNFα therapy (93); *second*, the two groups of new IGs and their pathways have been identified as novel therapeutic targets including: *i)* IGs and their pathways shared by upregulated IGs and suppressed IGs after deficiencies of PCs and regulators; and *ii)* IGs and the pathways upregulated after deficiencies of PCs and regulators; *third*, deficiencies of PCs and regulators increase inflammatory diseases in up-regulated IGs. The new problems need to be seriously considered when designing blockade therapies for PCs and regulators; one new mechanism has been identified for modulating the expressions of IGs carrying out the second wave of inflammation: PCs blockade can regulate the expression of ROS regulators and up-regulated ROS regulators can drive the upregulation of “suppressed IGs”. Taken together, we propose that multiple pathway convergent points can be new therapeutic targets for the future development of novel inflammation modulation therapies. The proof of principles demonstrated in the examination of inflammatory paradoxes performed in the current manuscript are far-reaching and can be extended to other fields of study such as cancers, infectious diseases such as COVID-19 and various pathologies.

One limitation of the current study is that due to the low throughput nature of verification techniques, we could not verify every result we identified with the analyses of high throughput data. We acknowledge that carefully designed *in vitro* and *in vivo* experimental models will be needed to verify the PCs and regulators deficiencies-upregulated IGs further and underlying mechanisms we report here. In addition, when -omics data with well-controlled clinical samples become available, we need to further verify and consolidate some of our new findings identified in mice here. Our findings nevertheless provide novel insights on the roles of upregulated IGs in the pathogenesis of inflammatory diseases, novel pathways underlying the multi-pathway convergent point suppression therapeutics models as well as new targets for the future therapeutic interventions for various inflammations.

## Data Availability Statement

All the datasets used in this study are publicly available. The analyzed results in this study are included within the article and the **Supplementary Materials**.

## Author Contributions

ML carried out the data gathering, data analysis and prepared tables and figures. JS, RZ, YSh, YSu, WY, JW, LuL, CD, CJ, FS, YL, KX, LiL, XW, XJ, and HW aided with analysis of the data. XY supervised the experimental design, data analysis, and manuscript writing. All authors contributed to the article and approved the submitted version.

## Funding

This work was partially supported by NIH grants to HW and XY.

## Conflict of Interest

The authors declare that the research was conducted in the absence of any commercial or financial relationships that could be construed as a potential conflict of interest.

## Supplementary Material

Supplementary Figure S1 The heatmap of diseases related to down-regulated innatomic genes from IPA analysis comparison.

Supplementary Table S1 Nine proinflammatory cytokine blocking monoclonal antibody therapies and clinical uses.

Supplementary Table S2 Ten additional inflammation paradoxes related to this study which have been reported.

Supplementary Table S3 The IGs and ROS regulators gene-lists used in this study.

Supplementary Table S4 The expression changes of housekeeping genes selected in this study.

Supplementary Table S5 The expression changes of up-regulated and down-regulated innatomic genes in deficiencies of proinflammatory cytokines and regulators. (*p*<0.05, ∣Log2FC∣>1)

Supplementary Table S6 The expression changes of up-regulated and down-regulated innatomic genes in Mab therapy microarrays. (*p*<0.05, ∣Log2FC∣>1)

Supplementary Table S7 IPA results of up-regulated innatomic genes in deficiencies of proinflammatory cytokines and regulators. (∣Z score∣> 2)

Supplementary Table S8 IPA results of down-regulated innatomic genes in deficiencies of proinflammatory cytokines and regulators. (∣Z score∣> 2)

Supplementary Table S9 Pathways from IPA results of up- and down-regulated innatomic genes in deficiencies of TFs. (∣Z score∣> 2)

Supplementary Table S10 IPA results of up- and down-regulated innatomic genes in anti-TNF therapy microarray.

Supplementary Table S11 The expression changes of ROS regulators in deficiencies of proinflammatory cytokines and regulators. (*p*<0.05, ∣Log2FC∣>1)

## Abbreviations

CVDs, cardiovascular diseases; IGs, innatomic genes (innate immune genes/regulators); PCs, proinflammatory cytokines/regulators; ROS, reactive oxygen species; Treg, regulatory T cells; IL-1β interleukin-1β; miRs: microRNAs; miR155, microRNA-155; KO, knock-out; MHO, metabolically healthy obesity; RA, rheumatoid arthritis; TNFα, tumor necrosis factor-α; Mab, monoclonal antibody; IL-6R, IL-6 receptor; DAMPs, danger associated molecular patterns; PAMPs, pathogen associated molecular patterns; NCDs, non-communicable diseases; NIH, National Institutes of Health; NCBI, National Center for Biotechnology Information; GEO, Gene Expression Omnibus; IPA: Ingenuity Pathway Analysis; GSEA, Gene Set Enrichment Analysis; IFNγ, interferon-γ; STAT1, signal transducer and activator of transcription protein 1; IKK2, Inhibitor of NFKB kinase subunit-β; TRAF6, TNF receptor associated factor 6; IRAK1, interleukin-1 receptor-associated kinase 1; PBMCs, peripheral blood mononuclear cell populations; Th1, type 1 T helper cells; HMGB1, high mobility group box 1; STAT4, signal transducer and activator of transcription 4; Erk1/2, extracellular signal-regulated protein kinases 1 and 2; Pyk2, proline-rich tyrosine kinase 2; CrkL, CRK like proto-oncogene; iCOS, inducible T-cell co-stimulator; NF-AT, nuclear factor of activated T cells; IRF, interferon regulatory factor; HGF, hepatocyte growth factor; JAK, Janus kinases; MIF: macrophage migration inhibitory factor; PAK, p21-activated kinase; PI3K, phosphatidylinositol-3-kinase; AKT, protein kinase B; cAMP, cyclic adenosine monophosphate; TFs, transcription factors; PD-1, programmed cell death protein-1; DC, dendritic cells; mtROS, mitochondrial ROS; MPO, myeloperoxidase; NES, normalized enrichment score; FDR, false discovery rate; NK, natural killer.
