# Supplementary material for: Approaching Inflammation Paradoxes—Proinflammatory Cytokine Blockages Induce Inflammatory Regulators
Source: Front Immunol. 2020 Oct 19;11:554301. doi: 10.3389/fimmu.2020.554301 (PMC7604447; doi:10.3389/fimmu.2020.554301)
Supplement: Supplementary Figure S1 — The heatmap of diseases related to down-regulated innatomic genes from IPA analysis comparison. [file Image_1.pdf]

Supplementary Material.

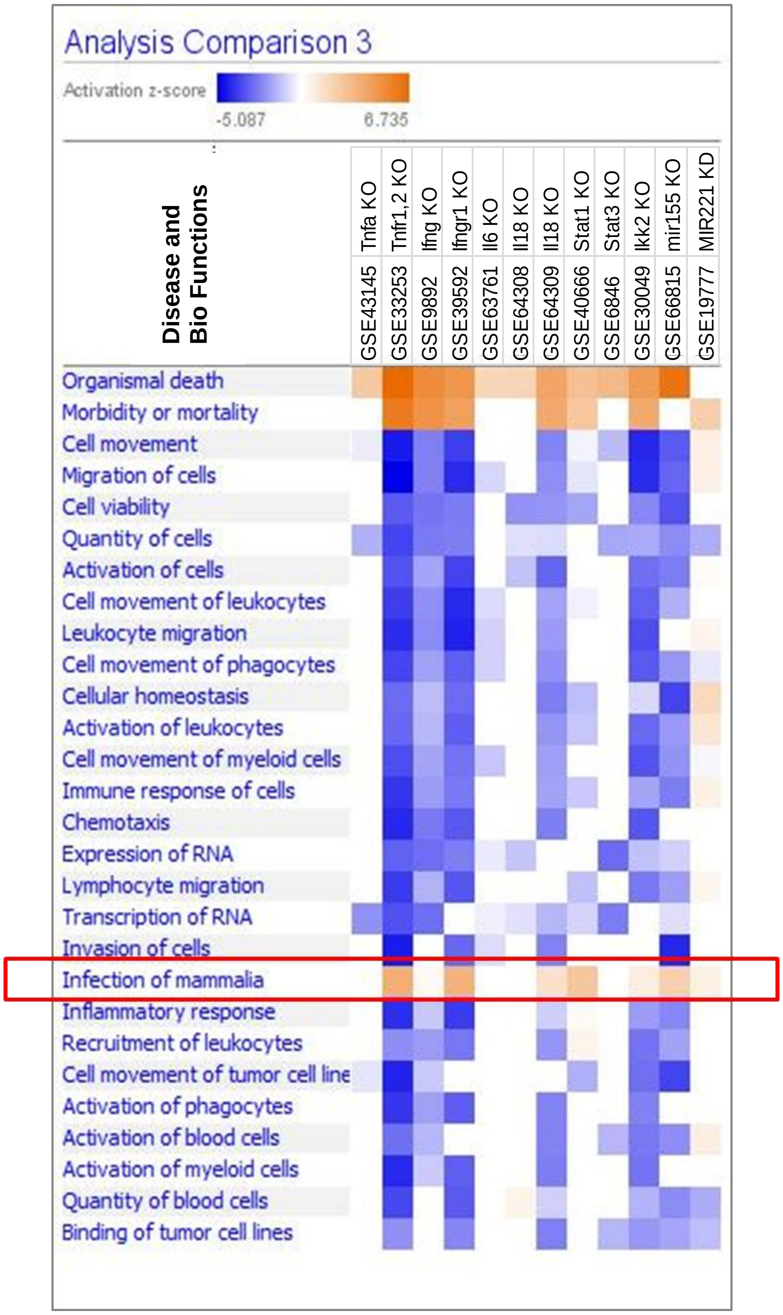

Supplementary Figure 1 The heatmap of diseases related to downregulated IGs from IPA analysis comparison. The result showed downregulated IGs were associated with increased infection of mammalia. KO, knock out; KD, knock down. (*P*.value<0.05)
