# Supplementary material for: Approaching Inflammation Paradoxes—Proinflammatory Cytokine Blockages Induce Inflammatory Regulators
Source: Front Immunol. 2020 Oct 19;11:554301. doi: 10.3389/fimmu.2020.554301 (PMC7604447; doi:10.3389/fimmu.2020.554301)
Supplement: Supplementary Table S1 — Nine proinflammatory cytokine blocking monoclonal antibody therapies and clinical uses. [file Table_1.pdf]

Table S1 Nine cytokine blocking monoclonal antibody therapies and their clinical uses were related to the proinflammatory cytokines in this study (Ref PMID: 31644151).

**Antigenic Target Cytokine**	**monoclonal antibody**	**Major Uses**
TNF	Adalimumab	Inflammatory bowel disease, Rheumatoid, psoriatic arthritis, Severe psoriasis
TNF	Certolizumab	Inflammatory bowel disease, Rheumatoid arthritis
TNF	Golimumab	Inflammatory bowel disease, Rheumatoid, psoriatic arthritis
IL1B	Canakinumab	Autoinflammatory diseases
IL6	Siltuximab	Castleman disease
IL6R	Tocilizumab	Rheumatoid arthritis
IL17A	Brodalumab	Plaque psoriasis
IL17A	Ixekizumab	Plaque psoriasis, Psoriatic arthritis
IL17A	Secukinumab	Plaque psoriasis, Psoriatic arthritis
