# Supplementary material for: Approaching Inflammation Paradoxes—Proinflammatory Cytokine Blockages Induce Inflammatory Regulators
Source: Front Immunol. 2020 Oct 19;11:554301. doi: 10.3389/fimmu.2020.554301 (PMC7604447; doi:10.3389/fimmu.2020.554301)
Supplement: Supplementary Table S2 — Ten additional inflammation paradoxes related to this study which have been reported. [file Table_2.pdf]

Table S2. Ten additional inflammation paradoxes related to this study have been reported.

No.	Paradoxes about inflammation	PMID
1	Mutated genes in TNF signaling, Il23, Il12 and integrin signaling are associated with inflammatory bowel disease.	28067908
2	Somatic mutations in blood cells as a mechanism of cardiovascular disease.	29420212
3	Modulating inflammation prevents cardiovascular diseases, but the effects on other chronic diseases, frailty, and disability are controversial.	30065258
4	Different haplotypes of the same single nucleotide polymorphisms from IL6 are associated with increased or decreased risk of rheumatoid arthritis.	25030201
5	Cytokine blockage therapies can increase the incidences of infections.	27856432, 16705109, 22751454
6	In Tnf KO tumor tissues, Tumor-promoting cytokines induced; Il18 or Il18 receptor KO mice led to hyperphagia, obesity and insulin resistance.	23975421, 16732281
7	Proinflammatory factor miR-155) deficiency in ApoE-/- mice exhibits improved atherosclerosis but results in obesity, non-alcoholic fatty liver disease.	27856635, 30369883
8	Obesity-related chronic low-grade inflammation attenuates sepsis-related mortality.	23594407, 27100585, 32064902
9	Increased inflammatory markers in Tsimane protected against obesity, type 2 diabetes, and cardiovascular diseases.	31448076
10	The increased proinflammatory Th1 type signals during implantation mammalian pregnancy curbed inflammatory reaction.	28850905
