# Supplementary material for: Approaching Inflammation Paradoxes—Proinflammatory Cytokine Blockages Induce Inflammatory Regulators
Source: Front Immunol. 2020 Oct 19;11:554301. doi: 10.3389/fimmu.2020.554301 (PMC7604447; doi:10.3389/fimmu.2020.554301)
Supplement: Supplementary Table S9 — Pathways from IPA results of up- and down-regulated innatomic genes in deficiencies of TFs. (∣Z score∣> 2) [file Table_9.pdf]

Table S9A. IPA results showed the significant pathways (｜Z score｜> 2) of up-regulated innatomic genes in proinflammatory related transcription factor KO microarray datasets. The data showed a total of five shared significant signaling pathway and 58 unique significicient signaling pathwayss of the up-regulated IGs in the proinflammatory related transcription KO microarrays. According to the IPA classification, Neuroinflammation Signaling Pathway, TREM1 Signaling, Dendritic Cell Maturation, Leukocyte Extravasation Signaling, etc, 17 cellular immune response signals (marked in bold) were activited by the up-regulated IGs.

No.	Significient signaling pathways	Nfkb subunit p65RelA-/- (GSE45755)	Stat1-/- (GSE40666)	Stat3-/- (GSE6846)
**1**	**Neuroinflammation Signaling Pathway**	↑	↑	↑
2	Cardiac Hypertrophy Signaling (Enhanced)		↑	↑
3	Osteoarthritis Pathway		↑	↑
4	Endocannabinoid Cancer Inhibition Pathway		↑	
**5**	**TREM1 Signaling**		↑	
**6**	**Dendritic Cell Maturation**			↑
7	B Cell Receptor Signaling			↑
**8**	**Leukocyte Extravasation Signaling**			↑
9	Tec Kinase Signaling			↑
**10**	**Production of Nitric Oxide and Reactive Oxygen Species in Macrophages**			↑
11	ERK/MAPK Signaling			↑
12	Integrin Signaling			↑
13	FGF Signaling			↑
14	NGF Signaling			↑
15	Cardiac Hypertrophy Signaling			↑
16	Synaptogenesis Signaling Pathway			↑
17	Mouse Embryonic Stem Cell Pluripotency			↑
**18**	**CD28 Signaling in T Helper Cells**			↑
19	EGF Signaling			↑
20	RANK Signaling in Osteoclasts			↑
21	Rac Signaling			↑
22	PDGF Signaling			↑
23	Type II Diabetes Mellitus Signaling			↑
**24**	**iCOS-iCOSL Signaling in T Helper Cells**			↑
25	Pancreatic Adenocarcinoma Signaling			↑
26	Ephrin Receptor Signaling			↑
27	Adrenomedullin signaling pathway			↑
28	LPS-stimulated MAPK Signaling			↑
**29**	**NF-κB Activation by Viruses**			↑
30	Cdc42 Signaling			↑
31	Signaling by Rho Family GTPases			↑
32	Cholecystokinin/Gastrin-mediated Signaling			↑
**33**	**IL-8 Signaling**			↑
34	p70S6K Signaling			↑
**35**	**PKCθ Signaling in T Lymphocytes**			↑
36	Phospholipase C Signaling			↑
37	Acute Phase Response Signaling			↑
38	Paxillin Signaling			↑
39	STAT3 Pathway			↑
40	HGF Signaling			↑
**41**	**p38 MAPK Signaling**			↑
**42**	**IL-6 Signaling**			↑
43	PPAR Signaling			↓
44	RhoGDI Signaling			↑
45	Ingenuity Canonical Pathways			↑

A total of 4 microarrays about proinflammatory-related transcription factors in supplementary Table 1B were analyzed. GSE30049 was not included because there were no significant (｜Z score｜< 2) pathways in it.

Table S9A. IPA results showed the significant pathways (｜Z score｜> 2) of up-regulated innatomic genes in proinflammatory related transcription factor KO microarray datasets (continued). The data showed a total of five shared significant signaling pathway and 58 unique significient signaling pathwayss of the up-regulated IGs in the proinflammatory related transcription KO microarrays. According to the IPA classification, Neuroinflammation Signaling Pathway, TREM1 Signaling, Dendritic Cell Maturation, Leukocyte Extravasation Signaling, etc, 17 cellular immune response signals (marked in bold) were activited by the up-regulated IGs. The detailed IPA results were showed in. (Continued)

No.	Significient signaling pathways	Nfkb subunit p65RelA-/- (GSE45755)	Stat1-/- (GSE40666)	Stat3-/- (GSE6846)
**46**	**Th17 Activation Pathway**			↑
47	Type I Diabetes Mellitus Signaling			↑
48	Lymphotoxin β Receptor Signaling			↑
49	IL-17A Signaling in Airway Cells			↑
50	Small Cell Lung Cancer Signaling			↑
51	Glioma Invasiveness Signaling			↑
**52**	**IL-7 Signaling Pathway**			↑
53	iNOS Signaling			↑
54	PAK Signaling			↑
55	Regulation of eIF4 and p70S6K Signaling			↑
56	April Mediated Signaling			↑
57	Renal Cell Carcinoma Signaling			↑
58	B Cell Activating Factor Signaling			↑
59	Insulin Receptor Signaling			↑
**60**	**IL-23 Signaling Pathway**			↑
61	Actin Cytoskeleton Signaling			↑
62	VEGF Signaling			↑
63	IGF-1 Signaling			↑
**64**	**IL-2 Signaling**			↑
65	Renin-Angiotensin Signaling			↑
66	GP6 Signaling Pathway			↑
67	Macropinocytosis Signaling			↑
68	IL-3 Signaling			↑
69	FLT3 Signaling in Hematopoietic Progenitor Cells			↑
70	PEDF Signaling			↑
71	Melanocyte Development and Pigmentation Signaling			↑
**72**	**Th1 Pathway**			↑
73	Relaxin Signaling			↑
74	eNOS Signaling			↑
75	Protein Kinase A Signaling			↑

Table S9B. IPA results showed the significant pathways (｜Z score｜> 2) of down-regulated genes in proinflammatory related transcription factors KO microarray datasets. Cellular immune response signals were marked in bold.

No.	Significient signaling pathways	IKK2-/- (GSE30049)	Stat1-/- (GSE40666)
1	Osteoarthritis Pathway	↓	
2	PPAR Signaling	↑	
3	Acute Phase Response Signaling	↓	
4	Type I Diabetes Mellitus Signaling	↓	
5	Cardiac Hypertrophy Signaling (Enhanced)	↓	
**6**	**Dendritic Cell Maturation**	↓	
**7**	**Production of Nitric Oxide and Reactive Oxygen Species in Macrophages**	↓	
**8**	**Neuroinflammation Signaling Pathway**	↓	
9	Endothelin-1 Signaling	↓	
**10**	**Interferon Signaling**		↓
**11**	**Activation of IRF by Cytosolic Pattern Recognition Receptors**		↓
**12**	**Systemic Lupus Erythematosus In B Cell Signaling Pathway**		↓

A total of 4 microarrays about proinflammatory-related transcription factors in supplementary Table 1B were analyzed. GSE6846 and GSE45755 were not included because there were no significant (｜Z score｜< 2) pathways in them.
